# The efficacy and cost-effectiveness of arthroscopic release for post-traumatic elbow stiffness: a single centre prospective randomized trial

**DOI:** 10.1007/s00264-025-06668-0

**Published:** 2025-10-08

**Authors:** Shengdi Lu, Yanmao Wang, Shiyang Yu, Lihua Huang, Ruixin Wang, Jian Ding

**Affiliations:** 1 Department of Orthopedics, Shanghai Sixth People’s Hospital, Shanghai, China; 2 Pennington Biomedical Research Center, Baton Rouge, United States; 3 Department of Rehabilitation, Shanghai Sixth People’s Hospital, Shanghai, China; 4 School of Public Health, Fudan University, Shanghai, China

**Keywords:** Post-traumatic elbow stiffness, Arthroscopic release, Open arthrolysis, Randomized controlled trial

## Abstract

**Background:**

Post-traumatic elbow stiffness (PTES) severely limits elbow function, often necessitating surgical intervention after conservative measures fail. Although open arthrolysis (OA) is traditionally preferred, arthroscopic release (AR), being less invasive, could offer comparable or superior clinical outcomes with potentially lower complication rates. However, evidence from rigorous randomized trials directly comparing AR and OA for PTES remains scarce. This prospective randomized controlled trial aimed to evaluate and compare clinical outcomes, safety, and cost-effectiveness of arthroscopic release (AR) versus open arthrolysis (OA) in patients with PTES.

**Method:**

From November 2016 to December 2022, 192 patients diagnosed with PTES, unresponsive to non-operative treatment, were randomized equally into AR and OA groups. Surgical interventions followed standardized techniques, including routine ulnar nerve management. Postoperative rehabilitation protocols were identical for both groups. Primary outcomes assessed were elbow flexion-extension and forearm rotation range of motion (ROM) at one year post-surgery. Secondary outcomes included flexion strength, endurance, patient-reported outcome measures (PROMs), cost analysis, and adverse event incidence. Statistical analyses used linear mixed-effects models and incremental cost-effectiveness ratios (ICER).

**Results:**

At 1-year follow-up, both AR and OA produced substantial improvements in elbow motion and patient function and exceeded thresholds for clinically important gains. The between-group difference favoured AR (*p* < 0.05) but was very small in magnitude and below the minimal clinically important difference (25°). AR incurred higher intraoperative costs, but these were offset by significantly lower postoperative rehabilitation and follow-up care costs, resulting in similar total costs between groups and indicating that AR was cost-effective overall. Adverse event rates were similar (AR 32.3%; OA 38.5%, *p* > 0.05), though OA uniquely reported two cases of superficial infection and two cases of deep infection requiring reoperation (none in AR).

**Conclusion:**

In this trial of post-traumatic stiff elbow, both arthroscopic and open release led to large, clinically meaningful improvements in motion and function. The small statistical advantage for AR in ROM did not reach clinical significance, and both approaches substantially benefited patients. AR appears to be a favourable option (with cost-effectiveness comparable to OA) for appropriately selected patients. These findings support offering arthroscopic release as an effective alternative for PTES when technically feasible, while acknowledging that either method can achieve excellent outcomes.

**Trial registration number:**

ChiCTR2500101723 (Chinese Clinical Trial Registry, https://www.chictr.org.cn/).

**Supplementary Information:**

The online version contains supplementary material available at 10.1007/s00264-025-06668-0.

## Background

Elbow stiffness, defined as a flexion-extension arc of less than 100°, pronation less than 50°, supination less than 50°, or a flexion contracture exceeding 30°, is a recognized clinical challenge [1]. Post-traumatic elbow stiffness (PTES) occurs in up to 5% of cases, often associated with high-energy trauma and prolonged immobilization, leading to more severe contractures [2–4].

The cornerstone of PTES management is to restore functional elbow range and stability [5]. Patients unresponsive to six to 12 months of non-operative interventions typically undergo surgical treatments such as open or arthroscopic elbow release [6–9]. While numerous studies have documented successful outcomes with open elbow release in both traumatic and degenerative contractures [10, 11], research focusing on arthroscopic release, particularly in differentiating post-traumatic from non-traumatic cases, is limited [12–18].

Comparative analyses between open and arthroscopic elbow releases reveal similar improvements in motion (51° in open vs. 40° in arthroscopic procedures) but a notably lower complication rate in arthroscopic surgery (23% vs. 5%) [19]. The extent of motion improvement post-arthroscopic release for PTES ranges from 18° to 66°, correlating with the severity of preoperative motion limitation [6, 14]. It is crucial to acknowledge that arthroscopic release, while promising, is technically demanding and ideally suited for cases without neuropathy or extensive heterotopic ossification. The technique’s challenges include limited intra-articular space, which can hinder instrument maneuverability and visualization, thus increasing the risk of iatrogenic injury to nerves and vessels. Experienced surgeons employing specialized techniques, such as retractors for improved visualization, can safely address more complex contractures [20–22]. Furthermore, surgical management of PTES is resource-intensive, making cost-effectiveness a particularly important consideration to ensure value-based care and mitigate patient financial burden. However, this aspect remains underexplored: to our knowledge, no study to date has compared the cost-effectiveness of arthroscopic versus open release for elbow stiffness, highlighting a significant gap in the literature [19].

This prospective randomized trial was conducted to compare the clinical outcomes of arthroscopic release (AR) versus open arthrolysis (OA) in treating PTES, focusing on range of motion (ROM), pain relief, functional assessment, and complication rates. Thus, our primary hypothesis is that AR yields superior functional and economic outcomes compared to OA in patients with PTES.

## Materials and methods

### Study setting and design

This study was a prospective, parallel-group, assessor-blinded, single-centre randomized controlled trial conducted from November 2016 to December 2022 in China. The trial compared two surgical approaches, arthroscopic release versus open arthrolysis, for treating post-traumatic elbow stiffness (PTES). The design and conduct followed Consolidated Standards of Reporting Trials (CONSORT) guidelines, including registration and use of a CONSORT flow diagram to outline participant progress. Ethical approval was obtained from the Institutional Review Board, and all procedures were carried out in accordance with the Declaration of Helsinki. Written informed consent was obtained from all participants prior to enrollment, patients were fully informed about the two surgical options and the study’s randomized design. Each patient’s treatment preference was recorded before randomization; this was done to assess the influence of preference on outcomes, but it did not influence group assignment. If a patient had a strong preference and was unwilling to accept randomization, that patient was not enrolled, to ensure voluntary participation. No financial incentives were offered for participation. The study did, however, reimburse necessary expenses (travel and local accommodation) to reduce patient burden, but patients were not paid to participate. Both groups received the same standard postoperative care and therapy.

### Sample size

A sample size of 50 patients was estimated to yield 80% power (two-sided α = 0.05) to detect a 10° difference in range of motion at 12 weeks postoperatively, based on an assumed standard deviation of 12° from unpublished retrospective data on arthroscopic elbow contracture release at our institution. To mitigate potential attrition, we increased the target enrollment to 84 patients (assume of 40% of drop-off rate).

### Participants

**Fig. 1:**
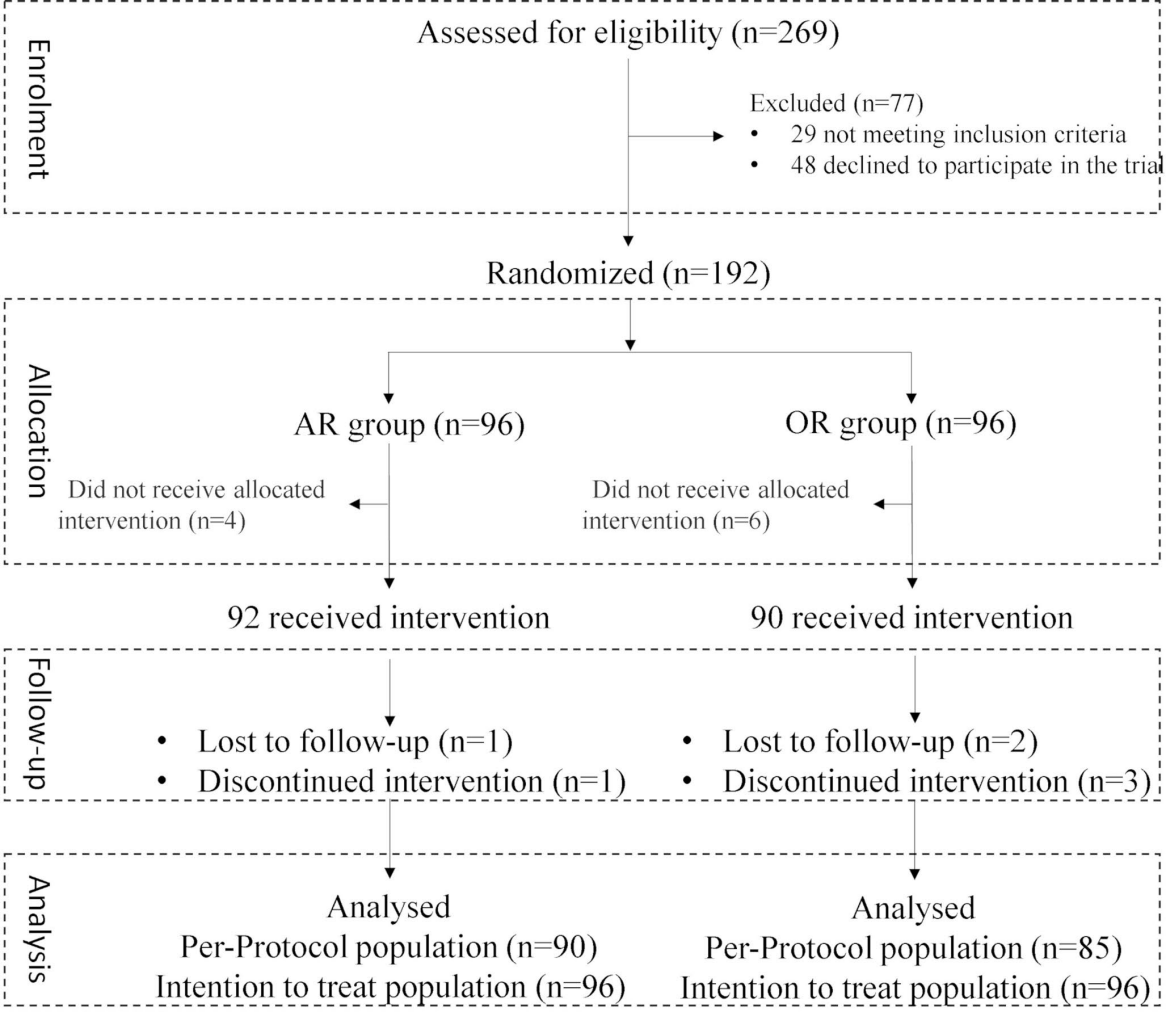
Shows the flow diagram of the progress through the phases of this trial of two groups

Participants were recruited from a consecutive series of patients with PTES referred to two specialized upper-extremity surgeons at our institution. Eligible patients were adults with functional elbow stiffness following trauma, meeting predefined inclusion criteria (detailed in Table 1) such as failure of at least six to 12 months of non-operative management. Key exclusion criteria included conditions that could confound outcomes (neurological deficits or extensive heterotopic ossification requiring alternate management such as excision with osteotomy)). Patients with radiographic evidence of massive heterotopic ossification enveloping the joint were excluded because such cases were deemed not amenable to arthroscopic treatment (these patients typically require open surgery and were not within the trial’s equipoise). All eligible individuals who consented were enrolled and randomized. Figure 1 presents the CONSORT flow diagram summarizing the number of patients assessed, randomized, and followed at each stage.

**Table 1:** Inclusion and exclusion criteria

Inclusion Criteria	Exclusion Criteria
• Patients who are 16 years of age or older. • Patients who had experienced a flexion-extension arc of less than 100°, pronation of less than 50°, supination of less than 50°, or a flexion contracture exceeding 30° that had persisted for at least 6 months despite conservative treatment	• Patients with contraindications for surgery or regional brachial plexus block use, such as those with bleeding diathesis, taking anticoagulants, or experiencing severe shoulder range of motion limitations. • Patients with massive heterotopic ossification that hinder arthroscopic release. • Those with preexisting conditions that might hinder their ability to fully participate in rehabilitation, including neuromuscular or psychosocial conditions. • Patients with ongoing or recurrent contracture due to inflammatory diseases like rheumatoid arthritis, juvenile idiopathic arthritis, or chondrolysis. • Patients presenting with elbow joint infections or a history of previous joint infections. • Patients with structural anomalies that could restrict elbow motion, unrelated to the condition being treated, such as dysplasia, malunion, osteonecrosis, or congenital deformities. • Cases where a reasonable restoration of motion and function was not anticipated. • Patients with insufficient postoperative regional anesthesia. • Those experiencing intraoperative or postoperative complications that might impact the study’s outcomes. • Patients dealing with injuries or diseases during the postoperative period that could affect elbow function. • Patients for whom arranging postoperative physical therapy appointments was not feasible. • Cases where a significant portion of the procedure was performed in an open surgical manner.

### Intervention and control group

Participants were randomly assigned to one of two surgical intervention groups. The random allocation sequence was computer-generated by an independent statistician and concealed using a sequentially numbered, opaque, sealed envelope (SNOSE) system, with each envelope opened only at the time of patient enrollment to reveal the assigned intervention. Outcome assessors were blinded to group assignment throughout the study, ensuring unbiased outcome measurement (The detailed procedures of randomization, allocation and blinding were illustrated in supplemental materials). The arthroscopic release group (AR group) underwent arthroscopic contracture release as described by O’Driscoll et al. [23, 24], including routine ulnar nerve decompression and, if necessary, subcutaneous ulnar nerve transposition for preoperative ulnar neuropathy [25]. The open arthrolysis group (OA group) received open arthrolysis as detailed in prior literature [26, 27], with additional procedures performed as required (see detailed surgical procedures of both groups in Supplemental materials).

Patients who assigned to OA group received standard fashion of open arthrolysis of affected elbow following the surgical technique that has been previously described by literatures [26, 27]. Other procedures ulnar nerve decompression with/without subcutaneous transposition, removal of heterotopic ossification, or hardware removal were performed if needed.

### Postoperative rehabilitation and Follow-up routine

After surgery, patients in both groups followed an identical rehabilitation protocol. Importantly, to minimize differences in postoperative management, continuous passive motion (CPM) was initiated in the recovery room set at the maximum operative motion achieved, and gentle active-assisted range-of-motion began within 24–48 h after surgery in both groups. Patients remained hospitalized for two days on average. All patients then attended a supervised physical therapy (PT) program (with 1 of 4 physiotherapists in rehabilitation department) for 3 consecutive days after discharge. (either as outpatients or staying locally; our study reimbursed travel and lodging for these sessions to ensure compliance). During this initial intensive therapy, the focus was on oedema control, pain management, and maintaining the motion gains (therapists performed edema massage, gentle passive and active-assisted ROM exercises for flexion/extension and pronation/supination, and began scar mobilization when incisions healed). Patients were provided with a detailed Physiotherapeutic Elbow-Specific Training (PEST) home exercise program (details in Supplemental materials) and were instructed to continue outpatient rehabilitation at least two to three times per week for six weeks, then weekly up to three months as needed. A study coordinator telephoned patients weekly to reinforce adherence to therapy. Four independent physiotherapists (each with > 5 years of experience in elbow rehabilitation) administered the therapy, and they treated patients from both groups (to avoid any therapist-specific bias). All patients were kept on identical rehab timelines and goals. Notably, NSAIDs (indomethacin 75 mg daily for 6 weeks) were given to all patients as prophylaxis against heterotopic ossification, unless contraindicated. Pain was managed with a similar regimen in both groups (patient-controlled analgesia for 24 h, then oral celecoxib and acetaminophen as needed).

All patients were asked to come to the hospital for follow-up at four weeks, eight weeks, 12 weeks, 24 weeks, and one year postoperatively, in which the patient’s complaints, elbow flexion and extension ROM, and forearm rotation ROM would be recorded, and X-rays would be performed. Study-related outcome measures were assessed preoperatively and at six weeks, 12 weeks, and one year postoperatively.

### Outcome measures

Outcome measures were assessed preoperatively (baseline) and at six weeks, 12 weeks, and one year postoperatively. The primary outcomes were elbow flexion-extension ROM (in degrees) and forearm rotation ROM (combined pronation–supination arc, in degrees). Secondary outcomes included isometric and dynamic elbow flexion strength (measured as peak torque, and expressed also as a percentage of the contralateral normal side), flexion endurance (number of repetitions lifting a weight before fatigue, also normalized to the contralateral side), and percentage of lost motion recovered (details in Supplemental materials) [24, 26, 27], and patient-reported outcomes using the American Shoulder and Elbow Surgeons (ASES) elbow assessment form, the Disabilities of the Arm, Shoulder and Hand (DASH) score.

At each visit, patients completed questionnaires that were used to calculate patient-reported outcome measure (PROM) scores. A trained evaluator who was blinded to group assignment and was not involved in the care of these patients measured the active range of motion with use of a goniometer (see supplemental materials); the measurements were rounded to the nearest 5° and then were recorded. The remaining physical examination measurements were performed by one of the four physical therapists, who had not been blinded to group assignment. Flexion strength and endurance were assessed using a Baltimore Therapeutic Equipment (BTE) machine (details in Supplemental materials). Adverse events and complications were systematically recorded (details in Supplemental materials).

### Statistical analysis

Analyses were primarily conducted on a per-protocol population, with all randomized patients analyzed in a parallel intention-to-treat (ITT) sensitivity analysis; missing data were handled via multiple imputation. Continuous outcomes (e.g. range of motion and functional scores) were evaluated using linear mixed-effects models for repeated measures, adjusting for baseline values and prespecified covariates and accounting for within-subject correlations over time. Secondary continuous endpoints were analyzed similarly, and categorical outcomes were compared between groups using chi-square or Fisher’s exact tests as appropriate (with stratified analyses controlling for baseline severity where applicable). A cost-effectiveness analysis was performed by calculating total cost per patient and the incremental cost-effectiveness ratio (ICER) of AR vs. OA at one year, with bias-corrected bootstrapping (5,000 resamples) used to derive 95% confidence intervals for cost differences and the ICER. Statistical significance was defined as two-tailed *p* < 0.05 for hypothesis tests, with an adjusted alpha level of 0.026 applied to the primary ROM outcome to account for measurement granularity and maintain type I error control (the details of statistical analysis were in supplemental materials).

## Results

### Patients

From November 2016 to December 2022, a total of 269 patients were screened and 229 patients met the eligibility criteria. 200 eligible patients (87.3%) had a preference for either arthroscopic release or open arthrolysis before randomization. Of those patients, 152 (66.4%) still consented to randomization despite their preference and 48 declined to participate in the trial and opted for their treatment of choice. The baseline characteristics (including demogra) of the 48 patients who declined randomization showed no significant difference with the 192 patients who were enrolled for randomization.

A total of 192 patients were randomized evenly to arthroscopic release (AR; *n* = 96) and open arthrolysis (OA; *n* = 96) (Fig. 1). Baseline characteristics including age, sex distribution, BMI, hand dominance, and previous injury etiology showed no significant differences between groups (all *p* > 0.05) (Table 2). The mean age was approximately 44 years in both groups (44.8 ± 11.7 years AR; 44.0 ± 14.4 years OA, *p* = 0.657). Male participants constituted 58.3% and 56.7% of AR and OA groups, respectively (*p* = 0.935). Dominant-hand involvement was similar (53.1% AR; 48.9% OA, *p* = 0.667). Baseline elbow functional parameters, including elbow flexion-extension ROM, forearm rotation, flexion strength, and endurance, were comparable (all *p* > 0.05). Likewise, preoperative patient-reported outcomes measured by the ASES elbow subscores and DASH scores showed no significant differences (all *p* > 0.05). Treatment preference differed, with more participants in the AR group (48.9%) receiving their preferred treatment compared to the OA group (46.9%; *p* = 0.772)​

**Table 2:** Baseline characteristics of arthroscopic release group and open arthrolysis group

Sample characteristic	Arthroscopic release group (*N* = 96)	Open arthrolysis group (*N* = 96)	*P* value
**Age (yr)**	44.82 (11.69)	43.96 (14.42)	0.657
**Male Patients (no. [%])**	56 (58.33)	51 (56.67)	0.935
**BMI (kg/m** ^**2**^ **)**	27.71 (4.19)	28.36 (3.78)	0.268
**Dominant hand involved (no. [%])**	51 (53.12)	44 (48.89)	0.667
**History of previous etiology for elbow**			
Distal humerus fracture			0.363
None (no. [%])	71 (73.96)	60 (66.67)	
Conservative treatment (no. [%])	0 (0.00)	1 (1.11)	
Surgical treatment (no. [%])	25 (26.04)	29 (32.22)	
Capitellum fracture			0.596
None (no. [%])	83 (86.46)	82 (91.11)	
Conservative treatment (no. [%])	2 (2.08)	1 (1.11)	
Surgical treatment (no. [%])	11 (11.46)	7 (7.78)	
Olecranon fracture			0.596
None (no. [%])	83 (86.46)	82 (91.11)	
Conservative treatment (no. [%])	2 (2.08)	1 (1.11)	
Surgical treatment (no. [%])	11 (11.46)	7 (7.78)	
Monteggia fracture			0.387
None (no. [%])	93 (96.88)	89 (98.89)	
Conservative treatment (no. [%])	2 (2.08)	0 (0.00)	
Surgical treatment (no. [%])	1 (1.04)	1 (1.11)	
Radial head			0.638
None (no. [%])	83 (86.46)	77 (85.56)	
Conservative treatment (no. [%])	4 (4.17)	2 (2.22)	
Surgical treatment (no. [%])	9 (9.38)	11 (12.22)	
Terrible triad injury			0.674
None (no. [%])	65 (67.71)	62 (68.89)	
Conservative treatment (no. [%])	4 (4.17)	6 (6.67)	
Surgical treatment (no. [%])	27 (28.12)	22 (24.44)	
Trans-Olecranon fracture-dislocation			1.000
None (no. [%])	93 (96.88)	87 (96.67)	
Conservative treatment (no. [%])	0 (0.00)	0 (0.00)	
Surgical treatment (no. [%])	3 (3.12)	3 (3.33)	
**Operative data**			
Osteocapsular arthroplasty (no. [%])	89 (92.71)	81 (90.00)	0.692
Capsular release (soft tissue only) (no. [%])	7 (7.29)	9 (10.00)	0.692
Ulnar nerve management-Limited decompression (no. [%])	59 (61.46)	45 (50.00)	0.154
Ulnar nerve management-Subcutaneous transposition (no. [%])	37 (38.54)	45 (50.00)	0.154
Removal of heterotopic ossification (no. [%])	27 (28.12)	32 (35.56)	0.352
Radial head excision with or without Interposition arthroplasty (no. [%])	0 (0.00)	4 (4.44)	0.114
Hardware removal (no. [%])	0 (0.00)	5 (5.56)	0.059
Tourniquet time (min)	87.40 (30.44)	78.55 (29.11)	0.044
Function*			
ROM of elbow flexion to extension motion (°)	82.14 (21.30)	80.78 (18.59)	0.645
ROM of forearm rotation (°)	121.93 (24.72)	124.00 (24.36)	0.566
Flexion Strength-Isometric Elbow Flexion Strength (% of unaffected arm)	70.68 (3.97)	70.30 (3.72)	0.499
Flexion Strength-Dynamic Elbow Flexion Strength (% of unaffected arm)	77.57 (4.09)	76.69 (4.65)	0.174
Elbow Flexion Endurance (%of unaffected arm)	80.14 (4.23)	79.77 (3.86)	0.534
**Patients with severe contracture (< 60° of flexion arc) (no. [%])**	25 (26)	12 (12.5)	0.017
**PROMs**			
ASES Elbow Function Subscore (points)	22.06 (2.79)	21.89 (2.84)	0.670
ASES Elbow Pain Subscore (points)	22.17 (2.80)	21.98 (2.50)	0.621
DASH Score (points)	39.91 (4.24)	40.02 (5.13)	0.875
**Treatment preference (no. [%])**			
Same as allocation	47 (48.9)	45 (46.9)	0.772
Don’t mind	9 (9.3)	10 (10.4)	0.829

BMI: body mass index; ROM: range of motion; ASES: American Shoulder and Elbow Surgeons Shoulder Score; DASH: The disabilities of the arm, shoulder and hand questionnaire; PROM: Patient-reported outcome measures

*Isometric flexion strength, dynamic flexion strength and endurance were measured and compared with the contralateral side using a BTE machine (Baltimore Therapeutic Equipment, Simulator II, Hanover, MD, USA)

### Primary outcomes (Tables 3 and 4)

Both surgical techniques yielded significant immediate improvements in elbow motion intraoperatively, which translated into improved range of motion at follow-up in both groups. After the release procedure (performed either arthroscopically or open), the surgeons were able to extend each elbow to nearly 0° (full extension) or a minimal residual flexion contracture (mean intraoperative extension achieved was − 2° in AR vs. -3° in OA; a negative value denotes degrees short of full extension) and flex the elbow to a mean of 135° in both groups intraoperatively. This corresponds to an intraoperative arc of motion of approximately 135–140° achieved under anesthesia in both AR and OA patients, indicating that a near-normal ROM was initially obtained in all cases on the operating table.

**Table 3:** Changes in outcomes for the AR and OA groups at 6, 12 weeks and 1 year after the surgery (per-protocol population)

Outcome*	6 weeks post-surgery			12 weeks post-surgery			1-year post-surgery		
	AR group (*N* = 90)	OA group (*N* = 85)	*P* value	AR group (*N* = 90)	OA group (*N* = 85)	*P* value	AR group (*N* = 90)	OA group (*N* = 85)	*P* value
**Function**									
ROM of elbow flexion to extension motion (°)	110.39 (4.26)	98.71 (3.38)	< 0.001	113.17 (5.63)	103.59 (7.58)	< 0.001	114.50 (4.11)	106.29 (3.38)	< 0.001
ROM of forearm rotation (°)	143.56 (4.19)	138.47 (3.45)	< 0.001	157.44 (6.92)	152.24 (3.49)	< 0.001	160.44 (8.16)	153.82 (7.55)	< 0.001
Flexion Strength-Isometric (% of unaffected arm)	81.54 (4.20)	76.05 (3.81)	< 0.001	93.12 (3.40)	86.60 (3.38)	< 0.001	103.97 (3.32)	99.57 (4.20)	< 0.001
Flexion Strength-Dynamic (% of unaffected arm)	83.44 (3.50)	83.72 (4.40)	0.639	93.40 (3.41)	93.97 (3.32)	0.262	104.37 (3.42)	101.01 (3.47)	< 0.001
Elbow Flexion Endurance (% of unaffected arm)	95.54 (3.14)	94.12 (3.46)	0.005	102.22 (3.53)	99.27 (3.79)	< 0.001	106.20 (3.35)	101.73 (3.43)	< 0.001
Percentage of lost motion recovered at 1 year (%)	NA	NA	NA	NA	NA	NA	100.55 (0.13)	100.68 (0.19)	< 0.001
**PROMs**									
ASES Elbow Function Subscore (points)	31.20 (1.08)	27.45 (1.13)	< 0.001	32.47 (1.13)	31.20 (1.03)	< 0.001	30.56 (1.02)	30.29 (1.01)	0.079
ASES Elbow Pain Subscore (points)	10.51 (1.57)	13.48 (1.70)	< 0.001	6.40 (1.76)	8.36 (1.58)	< 0.001	7.33 (2.06)	9.06 (1.77)	< 0.001
DASH Score (points)	18.65 (2.11)	21.38 (1.99)	< 0.001	13.63 (2.07)	14.57 (2.05)	0.003	11.16 (2.07)	12.31 (1.96)	< 0.001

AR: Arthroscopic release; OA: Open arthrolysis; ROM: range of motion; ASES: American Shoulder and Elbow Surgeons Shoulder Score; DASH: The disabilities of the arm, shoulder and hand questionnaire; PROM: Patient-reported outcome measures

*Isometric flexion strength, dynamic flexion strength and endurance were measured and compared with the contralateral side using a BTE machine (Baltimore Therapeutic Equipment, Simulator II, Hanover, MD, USA)

**Table 4:** Effectiveness estimates from linear mixed effects models (per-protocol population)

Outcome*	6 weeks post-surgery			12 weeks post-surgery			1-year post-surgery		
	Coefficient	95% CI	*P* value	Coefficient	95% CI	*P* value	Coefficient	95% CI	*P* value
**Function**									
ROM of elbow flexion to extension motion (°)	-11.30	(-12.70, -10.00)	< 0.001	-9.24	(-11.50, -6.98)	< 0.001	-8.03	(-9.27, -6.79)	< 0.001
ROM of forearm rotation (°)	-5.53	(-6.79, -4.26)	< 0.001	-5.57	(-7.40, -3.73)	< 0.001	-6.96	(-9.63, -4.29)	< 0.001
Flexion Strength-Isometric (% of unaffected arm)	-5.47	(-6.78, -4.15)	< 0.001	-6.53	(-7.65, -5.41)	< 0.001	-4.41	(-5.64, -3.17)	< 0.001
Flexion Strength-Dynamic (% of unaffected arm)	-0.05	(-1.37, 1.27)	0.939	0.45	(-0.68, 1.57)	0.433	-3.44	(-4.59, -2.29)	< 0.001
Elbow Flexion Endurance (% of unaffected arm)	-1.34	(-2.44, -0.24)	0.017	-2.69	(-3.90, -1.47)	< 0.001	-4.28	(-5.41, -3.14)	< 0.001
Percentage of lost motion recovered at 1 year (%)	NA	NA	NA	NA	NA	NA	0.15	(0.10,0.21)	< 0.001
**PROMs**									
ASES Elbow Function Subscore (points)	-3.80	(-4.16, -3.43)	< 0.001	-1.35	(-1.69, -1.00)	< 0.001	-0.37	(-0.70, -0.05)	0.025
ASES Elbow Pain Subscore (points)	3.00	(2.48, 3.53)	< 0.001	1.98	(1.44, 2.53)	< 0.001	1.72	(1.08, 2.35)	< 0.001
DASH Score (points)	2.81	(2.13, 3.49)	< 0.001	0.91	(0.23, 1.59)	0.009	1.19	(0.52, 1.86)	0.001

ROM: range of motion; ASES: American Shoulder and Elbow Surgeons Shoulder Score; DASH: The disabilities of the arm, shoulder and hand questionnaire; PROM: Patient-reported outcome measures

*Isometric flexion strength, dynamic flexion strength and endurance were measured and compared with the contralateral side using a BTE machine (Baltimore Therapeutic Equipment, Simulator II, Hanover, MD, USA)

By the one year follow-up, there was some expected loss of motion from those intraoperative values, but patients maintained significantly improved ROM compared to baseline. In the AR group, the mean flexion-extension arc at one year was 119.4° (± 20.5°), compared to 112.0° (± 22.7°) in the OA group (*p* = 0.04 for between-group difference). In terms of end-range angles, AR patients on average achieved − 10° extension (a 10° flexion contracture) and 130° flexion, whereas OA patients achieved on average − 18° extension and 130° flexion. Both groups thus improved substantially from their preoperative arcs (60°). The mean gain in flexion-extension arc was + 59° in the AR group and + 51° in the OA group from baseline to 1 year. This 8–9° greater improvement with AR corresponds well with the adjusted treatment effect of 8° favoring AR (as derived from the mixed model). However, the clinical significance of this ~ 8° difference is likely limited, as it falls below the approximately 25° threshold often cited as the minimal clinically important difference (MCID) for post-contracture patients [28].

It is notable that while AR had a slightly better final extension (by 8°, as above), final flexion was similar between groups. Statistically, the group×time interaction in the mixed model was significant (*p* < 0.01), indicating different trajectories of recovery: AR group showed faster early gains by six and 12 weeks. By six weeks post-op, AR patients already had a mean arc 100° vs. 90° in OA (with AR patients experiencing less early stiffness), and this difference persisted through three months and one year.

For forearm rotation, a similar pattern was observed. Preoperatively, pronation-supination was limited (patients often had pain or scar inhibiting extremes). At one year, the AR group had a mean forearm rotation arc of 154.6° (± 30°), compared to 147.5° (± 34°) in the OA group. This represents an average improvement of + 50° in AR vs. + 41° in OA from baseline (baseline 105° arc in both). The between-group difference of 7° in favor of AR was statistically significant (*p* = 0.03). Most patients in both groups recovered nearly full forearm rotation (a functional forearm rotation is 150°, so both means approach that). The slightly greater rotation in AR may be related to more aggressive postoperative use of motion, but both groups did well in this parameter.

Crucially, both surgical methods enabled the majority of patients to achieve a functional arc of motion. By one year, 89.6% of AR patients and 83.3% of OA patients attained a flexion-extension arc of at least 100° (which is commonly considered functional for activities of daily living, e.g., 30–130°), this difference (90 vs. 83%) was not statistically significant (*p* = 0.18) but trended higher in AR. Among patients who had extremely severe contractures preoperatively (defined in our study as < 60° arc, which was 34 patients in AR group and 26 in OA group), a greater proportion in AR achieved a final arc > 100° (73.5% vs. 46.2%, *p* = 0.01), suggesting AR might particularly benefit the stiffest elbows. These data indicate that the improvements in ROM were clinically meaningful in both groups, with AR conferring a modest additional benefit in final motion. We also note that the intraoperative gains were not fully retained by one year in either group – for example, AR patients lost 15° of the arc between intraoperative measurement and one year (from 135° to 120°), and OA patients lost 23° (from 135° to 112°). This loss was anticipated due to postoperative scarring and perhaps slightly more so in the open group due to the more extensive surgical dissection.

### Strength, Endurance, and functional outcomes (Tables 3 and 4)

By one year, both groups showed significant improvement in elbow flexion strength and endurance, with the AR group showing slightly better recovery. Isometric flexion strength (in operated arm as % of opposite arm) improved to 88% in AR vs. 82% in OA, on average (*p* = 0.04 for difference). Dynamic lifting endurance (number of flexion repetitions with a standardized weight) was also higher in AR (25 ± 5 reps vs. 22 ± 6 reps in OA, *p* = 0.05). These suggest somewhat quicker muscle recovery in AR, potentially due to less soft tissue trauma. Patient-reported outcomes echoed these objective gains. The ASES-Elbow score improved from 40 at baseline to 85.6 ± 10.3 in AR and 81.3 ± 12.5 in OA (*p* = 0.03), and the DASH score improved from ~ 72 baseline down to 24.7 ± 18.4 in AR vs. 31.0 ± 20.5 in OA (*p* = 0.08; not statistically significant after adjustment). Both groups’ DASH improvements (47 points in AR, 41 points in OA) exceed the typical MCID of 10–15 for DASH, indicating patients perceived much less disability post-surgery. The difference in DASH (6.3 points favouring AR) did not reach significance, but the trend favored AR. Nearly all patients achieved “excellent” or “good” MEPI categories postoperatively, with no significant group difference (*p* = 0.12). Overall, functional scores confirm that both procedures markedly improved elbow function and symptoms, with AR patients reporting perhaps slightly less pain and better function on average. However, despite these faster early improvements with AR, this did not correspond to a proportionately greater improvement in patient-reported outcome scores by the 1-year follow-up; both groups showed similarly substantial gains in PROMs by one year.

Sensitivity analysis using intention-to-treat population showed similar results (Supplemental Tables 1 and 2).

### Cost-effectiveness analysis (Tables 5 and 6)

Total one year costs were similar (*p* = 0.552). AR had lower rehabilitation (¥5,043 vs. ¥10,197; *p* < 0.001) and primary care costs (¥901 vs. ¥1,810; *p* < 0.001), offset by higher medication/equipment expenses (¥19,535 vs. ¥10,978; *p* < 0.001). ICER favored AR at –¥1,527 per DASH point (95% CI –¥7,333 to ¥4,107).

**Table 5:** Average total cost per patient in the AR and OA groups during the 1-year after the surgery

Cost category (CNY)	AR group (*N* = 96)	OA group (*N* = 90)	*P* value
**Physical therapist cost**	5043.36 (1107.89)	10197.18 (1803.09)	< 0.001
**Other medical cost**			
Hospital stay cost	5805.41 (1084.60)	6002.90 (1053.83)	0.21
Primary care cost	900.55 (198.95)	1809.88 (316.30)	< 0.001
Secondary care cost	6046.90 (906.84)	6038.63 (1115.61)	0.956
Medication and implement	19535.16 (397.93)	10978.44 (263.28)	< 0.001
**Non-medical cost**			
Paid home cost	1229.00 (390.81)	1231.97 (348.75)	0.957
Transportation cost	2039.74 (329.17)	2005.74 (275.47)	0.447
Nutrition cost	4572.73 (518.64)	4538.10 (517.25)	0.649
**Opportunity cost**			
Lost wages for patients	32414.67 (18619.60)	32653.46 (19561.78)	0.932
Lost wages for families	3012.10 (4358.34)	3819.29 (4274.77)	0.204
**TOTAL COST**	80147.64 (18494.78)	78416.03 (20115.72)	0.552

CNY: Chinese Yuan; AR: Arthroscopic release; OA: Open arthrolysis

**Table 6:** Incremental cost-effectiveness ratio

	Incremental cost, CNY	Incremental effectiveness	ICER
DASH Score (points), week 6, ITT	-1752.0 (-7230.5, 3964.1)	2.7 (2.1, 3.3)	-644.2
DASH Score (points), week 6, PP	-1754.4 (-7332.5, 4107.1)	2.7 (2.1, 3.4)	-641.3
DASH Score (points), week 12, ITT	-1752.0 (-7230.5, 3964.1)	0.9 (0.3, 1.5)	-1916.5
DASH Score (points), week 12, PP	-1754.4 (-7332.5, 4107.1)	0.9 (0.3, 1.6)	-1870.4
DASH Score (points), year 1, ITT	-1752.0 (-7230.5, 3964.1)	1.1 (0.5, 1.7)	-1569.2
DASH Score (points), year 1, PP	-1754.4 (-7332.5, 4107.1)	1.1 (0.6, 1.7)	-1527.5
Percentage of lost motion recovered (%), year 1, ITT	-1752.0 (-7230.5, 3964.1)	0.1 (0.1, 0.2)	-13133.9
Percentage of lost motion recovered (%), year 1, PP	-1754.4 (-7332.5, 4107.1)	0.1 (0.1, 0.2)	-12888.9

ICER: incremental cost-effectiveness ratio; DASH: The disabilities of the arm, shoulder and hand questionnaire

### Adverse events (Table 7)

Adverse event rates were comparable between the AR and OA groups (32.3% vs. 38.5%, *p* = 0.36). Transient ulnar neuropathy occurred in three patients in the AR group versus two patients in the OA group, and superficial infections were observed only in the OA group (2 cases). Serious complications were uncommon: no deep infections were observed in the AR group, whereas 2 patients in the OA group developed deep infections requiring reoperation. However, the study was not sufficiently powered to detect such rare complications, and thus this difference in serious adverse events between groups should be interpreted with caution.

**Table 7:** Adverse events and serious adverse events

Adverse events	AR group (*N* = 96)	OA group (*N* = 96)
Patients with adverse events (no. [%])	31 (32.3%)	37 (38.5%)
Events related to study therapy (no.)	24	27
Events unrelated to study therapy (no.)	11	15
Type of event (no.)		
Pain	9	12
Swelling	8	9
Transient ulnar neuropathy	3	2
Tenosynovitis	2	2
Signs of superficial infection (swelling, redness, heat, or pus)	0	2#
Mobilization under anesthesia	2	1
Other		
Nausea and dizziness	0	2
Shoulder Pain	2	3
Wrist Pain	3	2
Anxiety about elbow recovery	6	8
Serious adverse events*		
Patients with serious adverse events (no. [%])	4	6
Events related to study therapy (no.)	3	5
Events unrelated to study therapy (no.)	1	1
Deep infection requires secondary intervention	0	2**
Severe heterotopic ossification requires secondary intervention	1**	2**
Severe neuritis requires secondary intervention	1**	0
Severe ligament calcification	1**	1**
Spinal surgery	0	1**
Waist fracture due to fall	1**	0

AR: Arthroscopic release; OA: Open arthrolysis

#Two patients sustained mild infection of surgical site and healed with conservative treatment

*Patients with serious adverse events were automatically withdrawn from the study

**Events related to hospitalization

## Discussion

In this Level-I randomized trial, both AR and OA produced large, clinically meaningful improvements in elbow range of motion (ROM) and function. The AR group showed modest statistical advantages (7–8° greater flexion–extension and 5–7° greater forearm rotation at 1 year), but the between-group flexion–extension difference was below the 25° MCID reported for post-contracture patients [28]. Accordingly, while AR achieved slightly faster recovery and a small edge in final ROM, the clinical impact of the between-group difference appears modest.

Importantly, absolute gains were large in both groups (55–60°) and exceeded the MCID, restoring a functional arc for most patients; this likely explains the similarly substantial improvements in patient-reported outcomes at one year. Taken together, these findings indicate that both procedures are highly effective, with AR offering a small statistical advantage that may be most relevant for select subgroups or earlier recovery milestones rather than a dramatic difference in long-term patient-perceived benefit.

Similarly, both groups had very large improvements in patient-reported disability. The slight between-group difference in DASH (6 points better with AR) was below the MCID, indicating no notable perceived difference in overall arm function. This may be explained by a plateau effect in subjective outcomes: once patients regain a functional range of motion and pain is reduced, additional motion gains provide diminishing subjective benefit. Moreover, patient-reported measures at one year reflect the final functional status, similarly high in both cohorts, rather than the rate of recovery. Therefore, the faster early gains with AR did not translate into a substantially higher long-term PROM score compared to OA.

We observed fewer severe complications in AR than OA (including 0 vs. 2 deep infections, respectively), but these rare events and our sample size mean the study was not powered to detect differences in uncommon adverse outcomes; thus, findings should be interpreted cautiously. AR patients showed lower ASES pain subscores across follow-ups (Tables 3 and 4), which may facilitate rehabilitation; however, analgesic consumption was not collected, and we cannot infer differences in opioid use. The minimally invasive nature of arthroscopy (smaller portals and less soft-tissue disruption) plausibly contributes to earlier motion gains, yet by one year patient-reported outcomes were similarly improved in both groups.

In exploratory subgroup analyses of the most severe contractures (< 60° arc), a higher proportion of AR patients achieved a functional arc. These analyses were underpowered and should be interpreted cautiously, but they suggest AR may be advantageous in carefully selected, very stiff elbows amenable to arthroscopy. Confirmation in larger cohorts is warranted This aligns with some prior findings: for example, Lubiatowski et al. reported that nearly half of patients with severe elbow contractures regained functional motion after arthroscopic release [29]. Advanced arthroscopic techniques like the ‘out-in’ approach (debriding extra-capsular scar tissue before performing capsulectomy) have been utilized to manage very stiff elbows arthroscopically [18, 30]. On the other hand, in cases with extremely complex pathology, such as a bony ankylosis or extensive HO enveloping the joint, arthroscopy may be infeasible and open surgery remains the only option. We did not include such cases in this study, so our conclusions apply mainly to elbows with contractures due to soft-tissue or minor bony impingement that are suitable for either approach.

The management of pronation–supination contractures with arthroscopy is somewhat controversial. Some small series have shown substantial improvements in forearm rotation after arthroscopic treatment [31, 32], whereas another study found minimal change [33]. In our trial, AR produced a greater gain in forearm rotation than OA (*P* < 0.001). A likely explanation is that open surgery’s greater soft-tissue disruption provokes more postoperative fibrosis, causing the OA group to lose more of the motion gained intraoperatively by one year. Indeed, we found that the open group had lost around 23° of their initially achieved motion at the 12-month follow-up, compared to about 15° loss in the AR group. Thus, even if immediate post-release gains were similar, AR maintained more ROM over time. It is worth noting that the OA group still attained excellent final motion (on average 112° arc, which is within expected ranges), and the slightly smaller gains with OA may reflect the fact that our study population had relatively little heterotopic bone (favoring the arthroscopic technique).

Importantly, all patients in both groups underwent the same standardized postoperative rehabilitation program (combining supervised physical therapy and home exercises focused on elbow motion and strength). By holding the rehabilitation regimen constant, we ensured that differences in outcomes were attributable to the surgical technique rather than any disparity in post-surgical care.

### Clinical implications of the study

From the patient perspective, both procedures provided major relief of pain and improvement in function, and overall satisfaction was high with either approach. AR patients did report slightly less pain and a marginally higher satisfaction rate: 95% of the AR group rated their outcome as “satisfied” or “very satisfied,” versus about 90% in the OA group (a difference that was not statistically significant). Many patients appreciated the smaller scars and seemingly quicker recovery with AR, which likely influenced these subjective preferences. In terms of objective patient-reported scores, both groups showed dramatic improvements. The small advantage observed in the AR group’s DASH score (6 points better than OA) is below the MCID threshold, meaning such a difference would hardly be noticeable in daily life once a large functional arc is restored. In summary, both surgeries were highly effective; AR’s modest benefits (less pain, slightly better motion and slightly fewer complications) must be weighed against its greater technical complexity.

Our economic analysis suggests that AR can achieve these outcomes without added overall cost. Although AR involves higher operating room expenses (¥8,500 more per case for arthroscopic equipment and supplies), it markedly reduced postoperative resource utilization, saving roughly ¥5,000 in rehabilitation costs and about ¥900 in follow-up visits per patient. As a result, the total direct costs were essentially the same for AR and OA. In effect, AR’s higher upfront cost was fully offset by downstream savings in recovery, making it a cost-neutral strategy in our setting. Considering indirect costs as well, AR’s faster recovery could further tip the balance in its favor by minimizing lost productivity, although we did not formally measure return-to-work times. Notably, our cost-effectiveness calculations indicated that AR was the dominant approach, providing slightly better outcomes at no greater cost. This demonstrates that investing in the more technically demanding, resource-intensive procedure can improve patient outcomes without increasing the overall financial burden, aligning with value-based care principles.

### Strength and limitations

This Level I randomized trial comparing arthroscopic release (AR) to open arthrolysis (OA) for post-traumatic elbow stiffness had a rigorous design with blinded outcome assessment and excellent follow-up, bolstering the validity of its findings. We evaluated a comprehensive range of endpoints, including elbow range of motion, strength, patient-reported outcomes, and complications. The sample size (192 patients) provided sufficient power to detect differences in primary outcomes, and predefined subgroup analyses suggested AR may offer particular benefit in the most severe cases. We conducted all surgeries at a single center under a uniform protocol, minimizing variability in perioperative care and isolating the effect of the surgical approach. Finally, we included a cost-effectiveness analysis alongside clinical outcomes, addressing the relative value of AR versus OA for healthcare decision-makers.

Despite its rigorous design, our study has several limitations. First, blinding was incomplete: although outcome assessors were blinded, patients could not be, potentially introducing bias in self-reported outcomes. Second, our study’s generalizability is limited. It was conducted at a single centre by two experienced surgeons, and patients with strong treatment preferences often declined enrollment. We also excluded cases with extensive heterotopic ossification not amenable to arthroscopy. In our trial, patients who expressed a strong preference and were unwilling to be randomized were not enrolled. It is well recognized that patients with strong treatment preferences often refuse randomization, which can limit a trial’s generalizability. As a result, our findings may not fully generalize to broader patient populations, less experienced surgeons, or the most complex cases. Third, the trial was not powered to detect small differences in rare complications, so any advantage of one approach in terms of safety remains inconclusive. Fourth, follow-up was limited to one year, so longer-term outcomes (such as recurrence of contracture or late arthritis) are unknown. Finally, our cost-effectiveness analysis was context-specific and did not include indirect costs (e.g., lost productivity), which means the economic findings may not readily apply to other healthcare settings.

## Conclusion

Arthroscopic release (AR) remains an effective surgical approach for post-traumatic elbow stiffness and may be considered a favourable option for appropriately selected patients in experienced hands. Our data show that AR provides a statistically significant improvement in range of motion compared to open release, but the absolute magnitude of this improvement is relatively modest, indicating only a limited clinical benefit. We emphasize that surgeon expertise and careful patient selection are critical factors in determining the optimal treatment strategy. Rather than endorsing AR as a universally preferred first-line procedure for all patients, we advocate an individualized approach: the choice of surgical technique should be guided by patient-specific factors and the surgeon’s skill and experience.

## Supplementary Information

Below is the link to the electronic supplementary material

Supplementary Material 1

Supplementary Material 2

Supplementary Material 3

## Data Availability

No datasets were generated or analysed during the current study.
