# Supplementary material for: The efficacy and cost-effectiveness of arthroscopic release for post-traumatic elbow stiffness: a single centre prospective randomized trial
Source: Int Orthop. 2025 Oct 8;49(11):2671–83. doi: 10.1007/s00264-025-06668-0 (PMC12594727; doi:10.1007/s00264-025-06668-0)
Supplement: Supplementary file 1 — Supplementary Material 1 [file 264_2025_6668_MOESM1_ESM.docx]

**Table S1, changes in outcomes for the AR and OA groups at 6, 12 weeks and 1 year after the surgery (intention-to-treat population)**

| **Outcome*** | **6 weeks post-surgery** | | | **12 weeks post-surgery** | | | | **1-year post-surgery** | | | |
| --- | --- | --- | --- | --- | --- | --- | --- | --- | --- | --- | --- |
|  | **AR group (N=96)** | **OA group (N=96)** | **P value** | | **AR group (N=96)** | **OA group (N=96)** | **P value** | | **AR group (N=96)** | **OA group (N=96)** | **P value** |
| **Function** |  |  |  | |  |  |  | |  |  |  |
| ROM of elbow flexion to extension motion (°) | 110.57 (4.28) | 98.78 (3.38) | <0.001 | | 113.02 (5.69) | 104.11 (7.85) | <0.001 | | 114.53 (4.17) | 106.33 (3.50) | <0.001 |
| ROM of forearm rotation (°) | 144.27 (5.08) | 139.11 (4.72) | <0.001 | | 157.55 (6.88) | 152.78 (4.38) | <0.001 | | 160.26 (8.47) | 153.89 (7.41) | <0.001 |
| Flexion Strength-Isometric Elbow Flexion Strength (%of unaffected arm) | 81.93 (4.36) | 76.43 (4.06) | <0.001 | | 92.76 (3.65) | 86.32 (3.51) | <0.001 | | 103.94 (3.26) | 99.72 (4.32) | <0.001 |
| Flexion Strength-Dynamic Elbow Flexion Strength (%of unaffected arm) | 83.21 (3.58) | 83.60 (4.42) | 0.509 | | 93.56 (3.59) | 94.00 (3.44) | 0.396 | | 104.49 (3.62) | 100.81 (3.57) | <0.001 |
| Elbow Flexion Endurance (%of unaffected arm) | 95.42 (3.20) | 94.09 (3.40) | <0.001 | | 102.18 (3.46) | 99.43 (3.81) | <0.001 | | 105.79 (3.64) | 101.53 (3.52) | <0.001 |
| Percentage of lost motion recovered at 1 year (%) | NA | NA | NA | | NA | NA | NA | | 100.55 (0.13) | 100.69 (0.19) | <0.001 |
| **PROMs** |  |  |  | |  |  |  | |  |  |  |
| ASES Elbow Function Subscore (points) | 31.24 (1.06) | 27.64 (1.39) | <0.001 | | 32.53 (1.12) | 31.33 (1.17) | <0.001 | | 30.53 (1.01) | 30.34 (1.11) | 0.215 |
| ASES Elbow Pain Subscore (points) | 10.42 (1.56) | 13.34 (1.79) | <0.001 | | 6.43 (1.73) | 8.32 (1.60) | <0.001 | | 7.36 (2.03) | 9.04 (1.79) | <0.001 |
| DASH Score (points) | 18.54 (2.19) | 21.42 (2.00) | <0.001 | | 13.48 (2.18) | 14.55 (2.07) | 0.001 | | 11.12 (2.05) | 12.28 (2.00) | <0.001 |

AR: Arthroscopic release; OA: Open arthrolysis; ROM: range of motion; ASES: American Shoulder and Elbow Surgeons Shoulder Score; DASH: The disabilities of the arm, shoulder and hand questionnaire; PROM: Patient-reported outcome measures.

*Isometric flexion strength, dynamic flexion strength and endurance were measured and compared with the contralateral side using a BTE machine (Baltimore Therapeutic Equipment, Simulator II, Hanover, MD, USA)
