# Supplementary material for: The efficacy and cost-effectiveness of arthroscopic release for post-traumatic elbow stiffness: a single centre prospective randomized trial
Source: Int Orthop. 2025 Oct 8;49(11):2671–83. doi: 10.1007/s00264-025-06668-0 (PMC12594727; doi:10.1007/s00264-025-06668-0)
Supplement: Supplementary file 2 — Supplementary Material 2 [file 264_2025_6668_MOESM2_ESM.docx]

**Table S2, effectiveness estimates from linear mixed effects models (intention-to-treat population)**

| **Outcome*** | **6 weeks post-surgery** | | | | **12 weeks post-surgery** | | | | **1-year post-surgery** | | | |
| --- | --- | --- | --- | --- | --- | --- | --- | --- | --- | --- | --- | --- |
|  | **Coefficient** | **95% CI** | **P value** | **Coefficient** | | **95% CI** | **P value** | **Coefficient** | | **95% CI** | **P value** |  |
| **Function** |  |  |  |  | |  |  |  | |  |  |  |
| ROM of elbow flexion to extension motion (°) | -11.40 | (-12.70, -10.20) | <0.001 | -8.57 | | (-10.70, -6.39) | <0.001 | -8.03 | | (-9.25, -6.81) | <0.001 |  |
| ROM of forearm rotation (°) | -5.36 | (-6.90, -3.82) | <0.001 | -4.99 | | (-6.81, -3.16) | <0.001 | -6.86 | | (-9.44, -4.28) | <0.001 |  |
| Flexion Strength-Isometric Elbow Flexion Strength (%of unaffected arm) | -5.43 | (-6.76, -4.10) | <0.001 | -6.54 | | (-7.67, -5.41) | <0.001 | -4.17 | | (-5.37, -2.98) | <0.001 |  |
| Flexion Strength-Dynamic Elbow Flexion Strength (%of unaffected arm) | 0.14 | (-1.13, 1.40) | 0.833 | 0.48 | | (-0.63, 1.59) | 0.395 | -3.68 | | (-4.81, -2.55) | <0.001 |  |
| Elbow Flexion Endurance (%of unaffected arm) | -1.26 | (-2.32, -0.20) | 0.020 | -2.49 | | (-3.65, -1.33) | <0.001 | -4.17 | | (-5.31, -3.03) | <0.001 |  |
| Percentage of lost motion recovered at 1 year (%) | NA | NA | NA | NA | | NA | NA | 0.15 | | (0.10,0.20) | <0.001 |  |
| **PROMs** |  |  |  |  | |  |  |  | |  |  |  |
| ASES Elbow Function Subscore (points) | -3.59 | (-3.97, -3.20) | <0.001 | -1.21 | | (-1.57, -0.86) | <0.001 | -0.28 | | (-0.59, 0.03) | 0.077 |  |
| ASES Elbow Pain Subscore (points) | 2.93 | (2.42, 3.45) | <0.001 | 1.96 | | (1.43, 2.49) | <0.001 | 1.75 | | (1.13, 2.37) | <0.001 |  |
| DASH Score (points) | 3.04 | (2.37, 3.72) | <0.001 | 1.10 | | (0.43, 1.77) | 0.002 | 1.31 | | (0.67, 1.95) | <0.001 |  |

ROM: range of motion; ASES: American Shoulder and Elbow Surgeons Shoulder Score; DASH: The disabilities of the arm, shoulder and hand questionnaire; PROM: Patient-reported outcome measures.

*Isometric flexion strength, dynamic flexion strength and endurance were measured and compared with the contralateral side using a BTE machine (Baltimore Therapeutic Equipment, Simulator II, Hanover, MD, USA)
