# Supplementary material for: The efficacy and cost-effectiveness of arthroscopic release for post-traumatic elbow stiffness: a single centre prospective randomized trial
Source: Int Orthop. 2025 Oct 8;49(11):2671–83. doi: 10.1007/s00264-025-06668-0 (PMC12594727; doi:10.1007/s00264-025-06668-0)
Supplement: Supplementary file 3 — Supplementary Material 3 [file 264_2025_6668_MOESM3_ESM.doc]

**Supplemental Materials:**

**Method**

*Intervention and Control group*

Surgical Procedures for Post‑Traumatic Elbow Stiffness with Arthroscopic Release [1-5]

- Operating Room Setup: Perform the procedure under general anesthesia (often supplemented by regional nerve block for postoperative pain). Position the patient in the lateral decubitus position with the affected arm supported and a high arm tourniquet applied. This allows gravity to help clear fluids and gives ample room for instrument manipulation. All bony landmarks (olecranon, epicondyles, radial head) are marked, and the joint is insufflated with saline to distend the capsule and push neurovascular structures away.
- Ulnar Nerve Precautions: If the patient has pre-existing ulnar nerve symptoms or a flexion contracture >30°, first perform an open ulnar nerve decompression at the cubital tunnel (in situ release of the cubital tunnel retinaculum) before arthroscopy. In cases of established neuropathy, transposition of the ulnar nerve to an anterior subcutaneous position is recommended. Even in asymptomatic cases, many surgeons prophylactically release the ulnar nerve to prevent stretch injury during elbow manipulation, as routine decompression significantly reduces postoperative neuritis risk.
- Portal Placement and Joint Inspection: Begin with posterior compartment portals for safety. Insert the arthroscope through a posterocentral (straight posterior) portal in the olecranon fossa, this area is filled with fibrous tissue in a stiff elbow and is relatively avascular and free of major nerves, making it a safe entry point. Use a posterolateral “soft spot” portal (centered between olecranon, lateral epicondyle, and radial head) as the viewing portal and a direct posterior portal for working instruments. Maintain continuous saline inflow to keep the joint distended (often with a low-flow pump to minimize extravasation). Under arthroscopic visualization, perform a systematic diagnostic inspection of the posterior joint to identify tight bands, osteophytes, or loose bodies.
- Posterior Capsulectomy and Debridement: Perform the posterior arthroscopic release first. Use a motorized shaver and radiofrequency ablator through the posterior portals to debride scar tissue in the olecranon fossa, which enlarges the working space. Next, release the posteromedial capsule, this region is critical for extension and is close to the ulnar nerve. Keep the shaver’s blade facing the bone and work epiperiosteally (right against the capsule’s bony attachment) to safely detach the capsule while protecting the ulnar nerve behind it. Resect the fibrous posterior capsule and any adhesions around the posterior band of the medial collateral (ulnar collateral) ligament, which often tightens in a flexion contracture. Debride the posterolateral gutter and around the radial head as well; shaving the thickened proximal edge of the annular ligament may be necessary to restore full forearm rotation. Use a burr to excise posterior osteophytes, such as olecranon tip spurs or olecranon fossa osteophytes, that impinge extension. Throughout the posterior work, the arthroscope view is via the posterolateral portal while instruments work through posterior/central portals, ensuring a clear view of shaving near the ulnotrochlear joint line.
- Anterior Release and Osteophyte Removal: After completing posterior capsulectomy, shift to the anterior compartment. Create an anterolateral portal (just proximal to the radial head, lateral to biceps tendon) as the viewing portal, under direct visualization from a posterior scope if needed. Then establish the anteromedial portal using an inside-out technique with a switching stick or spinal needle from the anterolateral side. Through these anterior portals, remove anterior fibrous adhesions and any osteophytes blocking flexion, common sites are the coronoid tip and fossa and along the olecranon fossa’s anterior margin. Use a shaver and burr to clear the coronoid and radial fossae of bone spurs. Perform a careful anterior capsulotomy: incise the anterior capsule along the humero-ulnar joint line from medial to lateral, releasing it from its proximal attachment. Notably, maintain fluid distension as long as possible, keep the anterior capsule intact until all debridement is done, then cut it at the very end. This “capsular tent” technique preserves joint space and visualization during work and prevents early fluid extravasation.
- Dynamic Assessment and Adhesion Breakage: After both anterior and posterior structures are released, gently take the elbow through the full range of motion under anesthesia. This intraoperative manipulation helps break any remaining adhesions and tests the effectiveness of your release. Flex and extend the elbow to identify residual blocks; if a soft endpoint remains, use the arthroscope to find any uncut bands or osteophytes (a “dynamic arthroscopy” check). For example, trapped fibrous bands in the olecranon or coronoid fossae may become evident at end-range and can be trimmed. Continue alternating between debridement and motion until the elbow achieves a functional arc (ideally 0–130° or better) or no further improvement without risking stability. At this stage, confirm that collateral ligaments are intact (the joint should feel stable valgus/varus). If valgus instability is noted due to extensive posteromedial release, protect the area and consider capsular repair; however, a standard capsulectomy that preserves the ligament origin should not produce instability in extension.
- Heterotopic Ossification (HO) Considerations: Minor heterotopic ossification present in the capsule or fossae can be excised arthroscopically with a burr or osteotome if it is not too extensive. Small, peripheral HO fragments are removed like osteophytes. However, extensive HO (encasing the joint or bridging bone) is a relative contraindication for arthroscopic release. In such cases the arthroscopic view and instrument maneuverability are severely compromised. If intraoperative inspection shows unexpected extensive bone blocks, one should convert to an open procedure to safely excise the HO. In summary, use arthroscopy for cases with either no HO or only limited, accessible HO; reserve open arthrolysis for massive heterotopic bone formation or ankylosis.
- Closure and Post-release Protocol: Before concluding, perform a final check of the ulnar nerve (if it was decompressed, ensure it lies free of tension; if transposed, secure it subcutaneously). Achieve meticulous hemostasis. Arthroscopic portals are usually closed with a suture or Steri-Strips; a suction drain is generally *not* required for pure arthroscopy due to minimal incision size and fluid egress through portals. Note: Post-operative care (bracing and early motion) is critical but is not detailed here as per instructions. The key intraoperative goal is achieving a smooth, impingement-free arc of motion while avoiding iatrogenic nerve injury. Studies have shown arthroscopic release can yield motion gains comparable to open surgery (average 40–50° arc improvement) with low complication rates when these techniques and precautions are followed.

Surgical Procedures for Post‑Traumatic Elbow Stiffness with Open Arthrolysis [5-7]

- Operating Room Setup: Open elbow arthrolysis is also done under general anesthesia (with or without a regional block for postop analgesia). Position is usually lateral decubitus with the arm supported on an arm rest or bolster, allowing circumferential access to the elbow. Some surgeons use a supine position with the arm across the chest or on a hand table for a lateral approach – the approach dictates positioning. A tourniquet on the upper arm helps maintain a bloodless field. All relevant landmarks (medial/lateral epicondyles, olecranon, ulna border) are marked to guide incisions and protect nerves.
- Ulnar Nerve Management: Identify and protect the ulnar nerve early in the exposure. If a lateral approach is used exclusively, the ulnar nerve is still at risk of stretch once the contracture is released, so many surgeons will decompress it through a small separate incision or at least palpate and safeguard it. In an extensive posterior or medial approach, make the ulnar nerve the first structure to find: decompress the cubital tunnel by releasing Osborne’s ligament and mobilize the nerve out of the cubital sulcus. If the nerve is very adherent or elongated, transpose it anteriorly in a subcutaneous pocket to prevent post-operative palsy. This step is especially crucial in patients with preoperative ulnar neuropathy or long-standing flexion contractures.
- Exposure and Incision (Lateral Approach): The lateral column approach is the workhorse for open elbow release in most post-traumatic stiffness cases. Make a curved lateral skin incision centered at the lateral epicondyle and extending posteriorly over the olecranon tip (this curved or hockey-stick incision provides extensile access). Dissect through subcutaneous tissue, taking care to preserve or cauterize any superficial veins. Identify the interval for a Kaplan approach or an extensor-split approach: one option is to split the extensor digitorum communis (EDC) aponeurosis in line with its fibers (often along the interval between EDC and extensor carpi radialis brevis). Alternatively, some use the Kocher interval (between anconeus and extensor carpi ulnaris) if more posterior-lateral access is needed. Gently retract the extensor muscle bellies to expose the capsule and joint: the capitellum, radial head, and lateral distal humerus should come into view upon splitting the extensor mechanism. Throughout the dissection, protect the radial nerve (located anterior to the lateral epicondyle, in the brachioradialis/ECRL interval), staying on the supinator/extensor side of the lateral column minimizes risk. Also avoid detaching the lateral ulnar collateral ligament (LUCL) from the humerus; a true Kaplan interval is anterior to the LUCL, preserving elbow stability. Once the joint capsule is exposed on the lateral side, longitudinally incise or excise the thickened capsule to open the joint (an “open capsulectomy”). This lateral capsulotomy allows entry into the joint for further releases.
- Capsular Release and Debridement: Through the lateral incision, perform a comprehensive release of the elbow capsule. Work both anteriorly and posteriorly from the lateral side – indeed, using a lateral approach alone, surgeons can release both the anterior and posterior capsules in many cases. For the anterior release, the elbow is flexed and the surgeon elevates the brachialis muscle off the anterior humerus as needed to access the tight anterior capsule. The anterior capsule is then sharply cut or excised from the humeral condyle and the coronoid process. This may involve excising scar tissue in the coronoid fossa and removing any anterior osteophytes (for example, long-standing contractures often have a spur at the tip of the coronoid or along the trochlea). For the posterior release, extend the elbow and excise the thickened posterior capsule off the olecranon and olecranon fossa. If the posterior band of the medial (ulnar) collateral ligament is extremely tight and hindering extension, it can be partially released (split) to gain motion, this structure forms the floor of the cubital tunnel and is often a contracture tether in post-traumatic stiffness. Release it judiciously, balancing improved extension with elbow stability. All fibrous tissue and adhesions in the posterior compartment (olecranon fossa and gutters) are removed. Throughout the capsulectomy, maintain epiperiosteal dissection (staying right on bone) to avoid excessive bleeding and to protect muscles and nerves. By the end of this step, the ulnohumeral joint should be free of constricting soft tissue, allowing a wide arc of flexion–extension.
- Removal of Osteophytes and Blocks: Perform an osteocapsular arthroplasty by excising any bony prominences that block motion. Common impingements include a hypertrophic olecranon tip or ossified posterior band, which impedes full extension – remove these with an osteotome, rongeur, or high-speed burr. Likewise, remove anterior osteophytes such as a prominent coronoid tip spur or humeral fossa osteophytes that block flexion. Use imaging (fluoroscopy or preoperative CT) as a guide to ensure all bony blocks identified pre-op (e.g., in the coronoid or radial fossae) are addressed. Heterotopic ossification present within the capsule or around the joint is resected at this time: use sharp dissection to peel HO off the normal bone in a sleeve (staying in the plane between HO and cortex, i.e., periosteal resection). It’s important to resect HO completely and flush to the bone to minimize risk of recurrence. Any loose bodies or malunited fracture fragments in the joint (if present from the original injury) should be removed or shaved down now as well. By the end of this bony debridement, the joint should have no mechanical blocks to motion on either the anterior or posterior side.
- Addressing Muscle/Tendon Contractures: In longstanding elbow stiffness, not only the capsule but also musculotendinous units may adaptively shorten. Inspect the triceps in extension, a “stiff” triceps tendon or adhesions in the posterior compartment may limit flexion. If so, perform a triceps lengthening (e.g., a V-Y plasty or tongue incision in the triceps aponeurosis) to allow the elbow to flex more freely. Similarly, check the biceps and brachialis in full extension; while true biceps contracture is rare, extensive scarring in the anterior compartment can tether extension, which should have been addressed by capsulectomy. Ensure any fibrous bands in muscle are released. These soft-tissue releases (lengthenings) are done only as needed in cases of severe contracture, but they are essential to achieve maximal motion in certain patients. (For example, Nobuta et al. emphasized that lengthening a fibrotic triceps and excising periarticular HO were key to restoring motion in post-traumatic contractures.)
- Alternate/Additional Approaches (Medial or Posterior): Tailor the surgical approach to the pathology. In many cases, the single lateral approach is sufficient; however, for very severe contractures or certain patterns of stiffness, a combined approach may be needed. A medial approach can be added to address residual contracture on the medial side (especially if there is bulky HO or scarring around the medial epicondyle or ulnohumeral joint). This involves a small incision over the medial epicondyle, decompression or transposition of the ulnar nerve (if not already done), and release of the anterior bundle of the MCL or resection of medial osteophytes if they restrict motion. A posterior midline approach (with an olecranon osteotomy in extreme cases, though rarely needed for contracture alone) can provide direct access to both columns in complex cases. In a classic posterior approach, the triceps may be split or reflected (Bryan-Morrey approach) to expose the joint. The choice of approach depends on the location of the tightest tissues and any prior surgical incisions.
- Intraoperative Assessment: After completing the osteocapsular release, the surgeon performs a thorough range-of-motion check. Gently bring the elbow into full flexion – it should now flex to the limit of soft tissues (often beyond 130°, unless prevented by bulky muscle). Extend the elbow fully – ideally achieving 0° or a slight hyperextension if normal. Forearm rotation (supination/pronation) is also tested; if there is residual limitation, consider whether the radial head or proximal radioulnar joint is impinged (e.g., the annular ligament or interosseous membrane may be contributors, typically addressed only if needed). If forearm rotation is limited by structures like the annular ligament and a pathological finding (such as a healed radial head fracture or HO in the interosseous space) is identified, you may incise the annular ligament or address that specific issue. Palpate around the elbow during motion to feel any tight bands; for instance, the surgeon might detect a remaining posteromedial band, if so, they can release it now. Ensure the ulnar nerve glides freely through the range of motion, if any tethering or snapping is felt at the cubital tunnel, it indicates the nerve needs further mobilization or transposition. Also verify joint stability: varus/valgus stress should reveal intact collateral ligaments. (Minor laxity can occur if extensive capsular release was done, but gross instability is uncommon in isolated arthrolysis – if present, repair or augmentation of the ligament may be required.) Intraoperative fluoroscopy can be used at this stage to confirm removal of all bony blocks.
- Closure and Postoperative Plan: Irrigate the joint thoroughly to remove bone debris (especially important after burring osteophytes to prevent third-body wear). If a significant capsular excision was done, some surgeons will place a suction drain in the joint to evacuate hematoma and reduce swelling. Re-approximate any split muscle (e.g., repair the EDC split with absorbable sutures to restore the extensor mechanism). Close the fascia and skin in layers. A sterile compression dressing is applied, often with the elbow in an extended or neutral position to maintain the gains in extension. The end result of an open arthrolysis is an elbow free of scar tissue and impinging bone, capable of significantly improved motion. Open releases have shown good outcomes even in severe post-traumatic cases, with one study reporting an average flexion arc improvement from ~53° pre-op to ~95° post-op, albeit with diligent rehab and occasional minor residual loss at long-term follow-up. The meticulous surgical techniques described – adequate neurolysis, complete anterior/posterior capsulectomy, osteophyte and HO removal, and selective muscle lengthening – are all crucial to maximizing motion gains while minimizing complications in post-traumatic elbow stiffness surgery

*Randomization, Allocation and Blinding*

Participants were randomly assigned in a 1:1 ratio to either the AR group or the OA group. The random allocation sequence was generated by an independent statistician not involved in patient recruitment, surgery, or outcome assessment. Allocation concealment was maintained using a sequentially numbered, opaque, sealed envelope (SNOSE) system. After a patient consented to participate, the next envelope in sequence was opened to reveal the assignment. To minimize potential biases, patients were asked about their treatment preference (arthroscopic vs open) prior to randomization; this preference was recorded but did not influence group assignment. All surgical procedures were performed by two experienced elbow surgeons. The operating surgeon was informed of the assignment only at the time after arrangement of surgery and could not be blinded to the procedure type, but the use of two specialized surgeons for both techniques helped standardize surgical skill and technique across groups. Cross-over between interventions were allowed, and all patients received the surgery as allocated. Participants were analyzed in the groups to which they were randomized, in accordance with the intention-to-treat principle.

Blinding was incorporated for outcome assessment to reduce bias. Due to the nature of the interventions, blinding of the surgeon and patients was not feasible (the surgical approach and incisions inherently revealed the group). However, patients were asked to wear tailored close-fitting long-sleeved clothes so that the evaluators measuring the primary outcomes were kept blinded to group allocation throughout the study. These assessors were not involved in the clinical care of the patients and performed measurements in a separate setting to avoid unintentional unblinding. To further protect blinding, they were instructed not to inspect the surgical sites; this was intended to prevent noticing differences in scarring that could indicate whether a patient had arthroscopic portals or an open incision. The patients were likewise instructed not to disclose their treatment to the assessors. Blinded assessment was primarily applied to ROM measurements and patient-reported outcomes. Other examinations (such as strength testing on the BTE machine) were administered by physical therapists who, by necessity of conducting therapy, were aware of the surgical approach; these therapists were not part of the outcomes assessment team. The trial was therefore single-blinded (assessor-blinded) with respect to the primary and secondary outcomes.

*Outcome Measures*

1. American Shoulder and Elbow Surgeons-Elbow (ASES-Elbow) [8]: The ASES-Elbow is a standardized elbow evaluation developed by the Research Committee of the American Shoulder and Elbow Surgeons^1^. The patient self-evaluation form is divided into two subscales: pain and function. The pain subscale contains visual analogical scales (from 0 = no pain to 10 = worst pain ever) for pain evaluation. Scores on the ASES-Elbow pain subscale range from 0 to 50, with higher scores indicating worse pain. The function subscale contains questions relating to the function of the arm. The responses are scored on a four-point ordinal scale: 0 = Unable to do; 1 = Very difficult to do; 2 = Somewhat difficult; 3 = Not difficult. Scores on the ASES-elbow function subscale range from 0 to 36, with higher scores indicating better function.

2. Disability of Arm, Shoulder and Hand (DASH) Questionnaire [9]: The DASH Questionnaire is a standardized questionnaire which evaluates impairments and activity limitations, as well as participation restrictions for both leisure activities and work^2^. All items of DASH are scored with a five-point scale: 1 = no difficulty; 2 = mild difficulty; 3 = moderate difficulty; 4 = severe difficulty; 5 = unable. For each module, the sum of the responses produces a score, which then is transformed to obtain the DASH scores. This score ranges between 0 (no disability) and 100 (severe disability) for each domain. Therefore, a high DASH score indicates severe disability.

3. Percentage of lost motion recovered [10]: The relative improvement in elbow ROM at 1 year was defined as the percentage of lost motion recovered from baseline to 1 year. Recognizing that the normal arc of elbow motion is 145°, the change as a percent of lost normal motion that was recovered at 1 year was calculated from the formula:

[(𝑎𝑟𝑐 𝑜𝑓 𝑚𝑜𝑡𝑖𝑜𝑛 𝑎𝑡 1 𝑦𝑒𝑎𝑟) − (𝑝𝑟𝑒𝑜𝑝𝑒𝑟𝑎𝑡𝑖𝑣𝑒 𝑎𝑟𝑐 𝑜𝑓 𝑚𝑜𝑡𝑖𝑜𝑛)/145 − (𝑝𝑟𝑒𝑜𝑝𝑒𝑟𝑎𝑡𝑖𝑣𝑒 𝑎𝑟𝑐 𝑜𝑓 𝑚𝑜𝑡𝑖𝑜𝑛)] 𝑥 100%

4. Isometric flexion strength, dynamic flexion strength and flexion endurance: Isometric flexion strength, dynamic flexion strength and flexion endurance of the operative side were measured and compared with the contralateral side using a BTE machine (Baltimore Therapeutic Equipment, Simulator II, Hanover, MD, USA). All measurements were performed bilaterally first on the non-surgical side and then on the surgical side under the following protocol:

1. The BTE machine was adjusted to correct lever arm length and isometric flexion strength was measured first. With the elbow flexed at 90° and the hand grasping the lever arm in supinated position, patients were asked to flex the lever arm as hard as possible and hold for six seconds. Patients were asked to perform this procedure two more times for a total of three trials. The strength measurement reported is the mean of 3 trials with a coefficient of variation < 10%.
2. Dynamic flexion strength was assessed with 50% of the peak isometric flexion strength recorded in the previous step moving through full ROM as fast as possible for 10 seconds. No resistance was set in extension. Dynamic strength was recorded in Engals as a function of power (work/time) (1 Engal is the effort required to move a load of 1 inch-pound 1° in 1 sec).
3. Flexion endurance was assessed also with 50% of the peak isometric flexion strength moving through full ROM at a steady pace of 15/minute for 2 minutes. Endurance was recorded as a function of total work done at the end of 2 minutes.

In addition, starting from the first postoperative day and for 90 days postoperatively, patients completed a paper diary. To confirm completion of their diaries, patients received a reminder call every week for the 90 days by the trial coordinator. To discourage participants from dropping out of the study, each patient in each group received a remuneration of 1000RMB at the completion of the study.

*Adverse events*

We systematically recorded adverse events and complications throughout the study period (details in Supplemental materials). An adverse event in this study was defined a priori as any unfavorable or unintended symptom, sign, or complication temporally associated with the surgery that either required additional treatment beyond the routine postoperative care or negatively affected the patient’s recovery progress. This included typical surgical complications (infection, nerve injury, etc.) as well as any unexpected prolonged symptoms. Notably, pain or swelling were only recorded as adverse events if they were excessive or unusually prolonged such that additional interventions were necessary (for example, if pain beyond the standard expected level necessitated an extra clinic visit for evaluation or a change in pain medication, or if swelling necessitated an aspiration or splinting beyond standard care). Routine postoperative pain controlled by prescribed analgesics and moderate swelling resolving in the first few weeks were considered expected events and not counted as “adverse.” We distinguished serious adverse events (SAEs) as those that were life-threatening, resulted in hospital readmission or extension of hospital stay, required invasive intervention (such as a re-operation), or resulted in significant persistent disability. Examples of SAEs in our context included deep infections requiring surgical drainage, persistent nerve palsy at final follow-up, or any complication requiring hospitalization. Minor complications (non-serious adverse events) included issues like transient neurapraxia (nerve symptoms that resolved), superficial wound problems, or stiffness requiring a corticosteroid injection or bracing. Adverse events were recorded at three intervals: intraoperatively to discharge, discharge to 12-week visit (patients kept diaries and reported events), and 12 weeks to 1 year. All adverse events were adjudicated by an independent clinician. Patients who experienced a serious adverse event were withdrawn from the study’s therapy protocol and given appropriate care (though still included in analysis per ITT principles).

*Postoperative Rehabilitation and Follow-up Routine*

Patients were provided with a detailed Physiotherapeutic Elbow-Specific Training (PEST) home exercise program (Table 1) and were instructed to continue outpatient rehabilitation at least 2–3 times per week for 6 weeks, then weekly up to 3 months as needed.

Table 1, Physiotherapeutic Elbow-Specific Training (PEST)

| **Week (Post-op)** | **Status & Key Assessments** | **Rehabilitation Content** | **Home Exercises** | **Adherence & Adjustments** |
| --- | --- | --- | --- | --- |
| **Week 1  (Immediate Post-op)** | **Pain & Swelling:** High immediately post-op; monitor pain (VAS) and edema.  **Wound:** Surgical dressings intact, no infection signs; suture line healing (stitches in place).  **ROM:** Severely limited pre-op; post-release arc improved but still restricted by pain.  **Neurovascular:** Check ulnar nerve sensation (due to traction during arthroscopy). | **Goal:** Prevent re-stiffening and protect healing tissues.  **Early mobilization:** Begin gentle, pain-free elbow ROM within 24–48 hours post-surgery​.  Emphasize flexion in first 1–2 weeks if surgeon recommends​, while moving into extension only to tolerance.  **Therapy sessions:** Daily or every other day PT in this first week if possible. Use active-assisted and passive elbow flexion/extension exercises (gravity-assisted flexion, gentle extension stretch).  **Adjacent joints:** Active wrist, hand, and shoulder ROM to maintain mobility and reduce swelling​.  **Pain management:** Cryotherapy (ice packs) after exercises and compression to control swelling​. Physician may prescribe NSAIDs (e.g. indomethacin 75 mg daily for 6 weeks to prevent heterotopic ossification​) and analgesics.  **Manual therapy:** Very gentle joint mobilizations (Grade I-II) for pain relief; soft tissue massage to reduce edema. | **Frequent ROM drills:** Hourly short-duration flexion-extension exercises (e.g. 5–10 minutes each hour while awake) within pain limits.  **Self-assisted stretches:** Use the opposite hand to assist bending and straightening the elbow to the point of stretch (5–6 sessions/day, 10–15 reps).  **Hand/forearm exercises:** Stress-ball squeezes and wrist bending/rotation exercises 3–5×/day to maintain strength.  **Shoulder mobility**: Pendulum swings or gentle shoulder elevation exercises daily to prevent shoulder stiffness. | **Considerations:**  **Protection:** A light removable splint or sling may be used for comfort between exercise sessions but remove it several times daily for motion exercises​. Avoid prolonged immobilization.  **Pain control:** Ensure pain is managed (meds 30 minutes before PT) so the patient can participate in exercise​.  **Frequency:** Emphasize near-hourly home ROM; patient/caregiver education is critical to prevent recurrent stiffness.  **Monitoring:** Educate patients to watch for warning signs (excessive pain, swelling, tingling) and to do only **non-painful ROM** to avoid inflammation. |
| **Week 2** | **Pain:** Improving but still present at end ranges; continue to track VAS pain with movement.  **Wound:** Skin healing well; suture removal around day 10–14 if not absorbable​.  **Swelling:** Moderating; mild effusion may persist.  **ROM:** Gradual gains – aim to approach functional arc (e.g. ~30–130°) or better. Goal is full passive flexion by end of week 2 if tolerated​. Extension likely still limited; measure degrees for progress. | **Goal:** Regain as much ROM as possible while initiating muscle activation.  **Stretching & mobilization:** Continue passive and active-assisted ROM in all planes (flexion, extension, pronation, supination) 3–5× daily. Add gentle prolonged stretching at end-range: e.g. low-load long-duration extension stretches using a towel or gravity (several minutes per session)​.  **Joint mobilizations:** Grade III oscillations or distraction techniques for extension if needed (posterior glides for flexion, anterior glides for extension) – within tolerance​.  **Scar management:** Once incision healed (~2 weeks), begin scar massage and elbow soft tissue mobilization to prevent adhesions.  **Muscle activation:** Initiate gentle isometric strengthening (pain-free): e.g. isometric biceps/triceps contractions, and light resistance for wrist flexors/extensors​.  **Modalities:** Continue icing after therapy and consider heat before sessions to warm tissues (if swelling is minimal). | **Home ROM program:** Continue aggressive home ROM exercises (active-assisted) at least 4–5 times/day​. Use gravity-assisted elbow extension (lying supine with arm overhead) and gentle self-overpressure into extension (~2 min hold, 3–4× daily)​.  **Self-stretch**: Extension stretch using other hand or resting arm on a pillow with a gentle weight for a few minutes. Flexion stretches by using the opposite hand to push forearm toward arm.  **Isometrics:** At home, perform pain-free isometric contractions (hold 5 seconds, 10 reps) for biceps, triceps, and forearm muscles, 2–3× per day.  **HEP log:** Patient keeps a log of daily exercise frequency to ensure compliance. | **Considerations:**  **No ROM restrictions:** Post-release, motion is allowed as tolerated (no structural repair to protect)​. Encourage reaching end-ranges but avoid aggressive force that causes sharp pain.  **Adherence:** The second week is critical – stress the importance of daily stretching (the major ROM gains are typically achieved in the first 4–6 weeks​).  **Customization:** If extension lags significantly behind flexion, consider a static progressive extension splint at night or dynamic brace to assist (optional, if standard exercises aren’t yielding gains)​.  **Therapy frequency:** 2–3 PT sessions/week to supervise stretching intensity, adjust techniques, and maintain motivation​. |
| **Week 3** | **Pain:** Generally mild at rest, moderate at end-range stretch; may still need occasional pain meds before therapy.  **ROM:** Improving; patient may have achieved functional flexion (~130°) and working on terminal extension (perhaps ~15–20° short of full). Pronation/supination nearly full if they were limited.  **Strength:** Slight atrophy of biceps/triceps from pre-op disuse; beginning to activate muscles. Grip strength improving if exercised.  **Functional:** Patient can do light ADLs (feeding, dressing) with less difficulty as ROM improves, but heavy tasks still limited. | **Goal:** Transition to active movements and begin light strengthening while continuing to improve ROM.  **Active ROM:** Progress to active-assisted ➔ active ROM in elbow flexion/extension as pain allows. Use active gravity-eliminated exercises (e.g. sliding arm on a table) progressing to against-gravity motions.  **Strengthening:** Initiate light isotonic strengthening for the elbow this week​. For example, begin biceps curls and triceps extensions with light dumbbells or resistance bands (e.g. 0.5–1 kg) for high-rep, low-load sets. Include forearm pronation/supination strengthening with light resistance (e.g. hammer rotations).  **Continued stretching:** After warm-up, continue focused end-range stretching – hold each stretch 30+ seconds. If lacking extension, therapist may apply prolonged passive stretch or use contract-relax techniques to gain last degrees.  **Shoulder & scapula:** Add scapular stabilizer exercises (like scapular pinches, serratus punches) and shoulder strengthening (rotator cuff isometrics) to support the overall arm function​.  **Neuromuscular control:** Begin **proprioceptive exercises** (e.g. elbow joint positioning drills, gentle closed-chain weight shifts) to re-integrate the elbow in functional patterns. | **Strength HEP:** Light dumbbell or Theraband exercises for elbow flexion/extension at home 3×/week (e.g. 2 sets of 15 reps at low weight). Maintain good form without pain.  **Stretching:** Continue daily ROM stretching routine – now emphasizing any remaining tight motion (often extension). For example, use a doorframe or wall to gently push the elbow into more extension stretch 2–3× daily (30–60s holds).  **Functional use:** Patient encouraged to use the arm in ADLs – e.g. grooming, eating, typing – to promote active functional ROM (within pain limits).  **Aerobic exercise:** Introduce lower-impact cardio that involves arms without heavy load (e.g. stationary bike or walking, gently swinging arms) to improve circulation and endurance. | **Considerations:**  **Weaning support:** By week 3, the sling is usually discontinued as comfort allows​, so the patient should try to keep the arm out of the sling to use it naturally.  **Pain management:** If stretching is very painful, short-term use of heat before exercise or analgesics can help. Emphasize that some discomfort is expected during stretching, but severe pain must be avoided​.  **Progression:** If ROM gains are slowing, therapists may increase intensity of mobilizations or add techniques like contract-relax. Conversely, if ROM is nearly full, shift more focus to strengthening while maintaining flexibility.  **Monitoring:** Continue weekly ROM measurements. By end of Week 3 or 4, aim for nearly full flexion and <10° extension lag if possible. Individual progress will vary; rehab plan may be adjusted accordingly. |
| **Week 4** | **Pain:** Minimal at rest; aches after therapy. Pain mainly at end-range extension if any.  **ROM:** Near full flexion likely (within a few degrees of opposite side). Extension still improving (perhaps ~10° short of full). Forearm rotation should be full or nearly full.  **Strength:** Elbow and forearm strength still ~30–50% weaker than the uninvolved side due to prior disuse but improving with exercise.  **Function:** Patient performing most light ADLs independently. Some difficulty with tasks requiring heavy lifting or end-range elbow extension (e.g. pushing up from a chair). | **Goal:** Achieve full elbow ROM and increase muscle strength/endurance.  **ROM & flexibility:** Continue aggressive end-range stretching to achieve full extension. May incorporate prolonged stretches (e.g. using a dynamic extension splint for 30 minutes if available, or therapist-applied 5-minute terminal stretch). Joint mob**s** as needed to address any remaining capsular tightness.  **Strength training:** Progress strengthening intensity: increase resistance slightly (e.g. move from 1 kg to 2 kg dumbbells as tolerated) for elbow flex/ext and forearm exercises. Begin resisted forearm supination/pronation (e.g. with light hammer or Theraband). Add shoulder strengthening (light dumbbell shoulder presses, rows) to integrate elbow use with shoulder.  **Endurance exercises:** Introduce light endurance training for the arm – e.g. UBE (arm cycle) with low resistance for 5–10 minutes​ to build tolerance for sustained activities.  **Functional movement:** Start gentle closed-chain exercises if appropriate: wall push-ups or table push-ups (partial weight) to encourage elbow extension under load (ensure no instability or pain).  **Manual therapy:** If scar tissue or myofascial tightness is palpable, continue soft tissue mobilization and begin more vigorous scar massage to mature the scar. | **Strength & endurance:** Home program now includes strength exercises 3–4 days/week (with a rest day between to allow recovery). Encourage higher repetitions (15–20 reps) to build endurance before focusing on pure strength.  **Stretch maintenance:** Continue daily stretching – even if full ROM is reached, keep stretching end-ranges to maintain it. For example, do a prolonged evening stretch into extension (using a weight on the wrist or lying face-up with arm overhead).  **Functional practice:** Patient to practice functional tasks at home that require elbow motion: e.g. reaching to a high shelf (elbow extension), lifting light objects (e.g. 1-2 kg) from floor to table (elbow flexion).  **Heat/ice as needed:** Use a heating pad before stretching at home if tight, and ice after exercise if swelling or soreness occurs. | **Considerations:**  **Milestone:** By ~4 weeks, many patients achieve full or nearly full passive ROM​. If patient has full ROM, shift goal to maintaining it and focusing on strengthening. If not, intensify stretching now before scar maturation makes gains harder.  **Adaptation:** Increase therapy exercises difficulty gradually (e.g. add slight resistance or new angles) to avoid plateau. But ensure no joint instability or pain with advanced exercises; if present, regress and strengthen more gradually.  **Patient education:** Reiterate that improvement slows over time – highest gains occur in early weeks​. Encourage patience and persistence, as even small weekly gains are meaningful.  **Clinical check:** Follow up with surgeon around 4-6 weeks to assess progress; the surgeon will expect substantial motion gains by now. Ensure communication about any concerns (e.g. abnormal pain or if extension still >30° limited). |
| **Week 5–6** | **Pain:** Generally low pain levels; may have mild soreness after exercises, but no significant pain during daily use.  **ROM:** Full flexion achieved; working on last few degrees of extension – patient may still lack ~5–10° (almost full straightening).  **Strength:** Improving; able to lift moderate light objects. By end of Week 6, expect ~70% strength return in elbow flexors/extensors, depending on pre-surgery muscle condition.  **Function:** Most ADLs pain-free. Can lift lighter groceries, perform personal care. Still regaining confidence in heavy or fast movements. | **Goal:** Solidify full ROM and advance to moderate strengthening and functional movements.  **Strength progression:** Moderate resistance strengthening now. Increase weight or resistance band level for biceps/triceps and wrist work (aim for fatigue at ~10–12 reps). Include eccentric exercises (e.g. slow lowering of weights) to build control. Forearm and grip strengthening with putty or grippers can be intensified.  **Dynamic exercises:** Begin more dynamic and multiplanar movements: e.g. light medicine ball tosses (two-handed initially) to engage elbow in coordination with shoulder. Incorporate PNF patterns diagonally involving the elbow (e.g. D2 flexion/extension patterns) to improve neuromuscular control​.  **Functional training:** **Simulate functional tasks** in clinic – e.g. reaching and lifting tasks, gentle pushing/pulling (light theraband rows, light bench press motions). If patient’s goals involve sports or manual work, tailor exercises (for example, mimic hammering motion, or throwing motion without weight).  **End-range focus:** If a slight extension deficit remains, continue end-range joint mobilizations and consider static progressive splinting at night to close the gap​.  **Endurance:** Increase cardiovascular exercise involving the arms: e.g. elliptical or light swimming movements (if wounds fully healed) to build endurance of the upper limb. | **Strength HEP:** Continue strengthening at home or gym. By week 6, patient can perform home strengthening 4–5×/week with gradually heavier resistance as tolerated (e.g. 2–3 kg weights for elbow exercises if able). They should incorporate both flexion/extension and forearm rotations.  **Stretching:** Nightly long-duration stretch for extension if any deficit remains (e.g. wear a static splint or use a weight on extended arm for 10 minutes). If full extension is achieved, still perform a brief stretch daily to maintain it.  **Functional use:** Encourage more use of the arm in household chores (light cleaning, carrying small loads) and work tasks (if applicable, with therapist guidance on safe techniques). Gradually reintroduce activities like driving (typically allowed ~6 weeks if pain-free and adequate control).  **PT homework:** If in a work-conditioning or work-hardening stage, follow specific drills provided by therapist to do on non-PT days. | **Considerations:**  **Phase transition:** By 6 weeks, the “acute” rehabilitation phase is ending​. The elbow should have minimal pain with motion and nearly full ROM, allowing a transition to more vigorous strengthening.  **Criteria to progress:** If patient has <10° extension loss and no swelling, they can safely advance strengthening​. If not, continue focused stretching before heavy strengthening.  **Patient confidence:** As strength returns, patient may start using the arm more normally. Emphasize proper body mechanics and avoiding compensatory movements (e.g. shoulder hiking) during tasks.  **Clinical:** If by 6 weeks significant stiffness persists (e.g. >30° motion loss), re-evaluate; more aggressive interventions (like dynamic splinting or a steroid injection) might be considered by the physician​. In most cases, however, continued therapy is pursued up to 12 weeks before such measures​ |
| **Week 7–8** | **Pain:** Minimal; typically only occasional soreness after heavy exercise. No rest pain.  **ROM:** Full elbow extension and flexion should be achieved or very close by this stage (0–140° or patient’s max). Pronation/supination full.  **Strength:** Near-normal for daily activities. Perhaps ~80% of contralateral side’s strength; remaining deficit in high-force or endurance tasks.  **Function:** Patient can perform most daily tasks normally. Possibly cleared for light work duties. Working on more demanding activities (sports, heavy labor tasks) as needed. | **Goal:** Restore full strength and begin higher-level functional training.  **Advanced strengthening:** Progress to heavier resistance and plyometric exercises as appropriate. For example, introduce push-ups (modified to full weight-bearing as tolerated), weighted ball throws or dribbling, and rapid elbow flex/extension drills to develop power. Ensure proper form and no elbow instability.  **PNF & agility:** Continue PNF patterns with increased resistance and speed. Add agility drills for the arm (for example, ladder drills for the hand or catching/light tossing to challenge elbow reaction and coordination).  **Functional simulation:** Incorporate work- or sport-specific exercises. If the patient is an athlete (e.g. racquet sports, baseball), begin sport-specific motion drills (shadow swinging a racket, mock throwing drills at submaximal intensity). If a manual worker, simulate lifting/carrying, using tools, etc., under supervision.  **Endurance:** Increase endurance challenges – e.g. longer duration arm bike or swimming laps, to ensure the elbow can tolerate sustained activity.  **Proprioception:** Add advanced proprioceptive training such as weight-bearing on an unstable surface (e.g. hands on a BOSU or wobble board) to enhance joint stability. | **Strength & power HEP:** Patient continues strengthening but can now include compound movements at home or gym (e.g. light dumbbell bench presses, overhead presses, kettle bell exercises) that involve the elbow. Perform these 2–3× per week, mixed in with prior elbow-specific exercises.  **Stretching:** At this stage, formal stretching can be reduced to a maintenance routine (e.g. brief stretches before and after workouts) if full ROM is already attained. If any tightness recurs, resume daily stretching as needed.  **Skill practice:** Encourage practice of specific skills relevant to patient’s goals outside of therapy. For example, gentle basketball free-throw practice, golf putting, or other low-stress practice that engages the elbow joint.  **Aerobic fitness:** If patient enjoys activities like swimming or biking, they can ramp up duration/intensity as cleared (these also help elbow endurance). | **Considerations:**  **Return to work/sport:** Clearance for work or sports is individualized. Many patients can start light sports or job tasks by ~8-12 weeks post-op, with full return by 3–6 months​. Ensure the patient demonstrates adequate strength and ROM required for their specific activities before full return.  **Joint protection:** Instruct on any necessary bracing or supports if returning to high-risk activities (though generally not needed if strength is recovered and ROM is full).  **Adaptive timing:** Some patients may reach this advanced phase sooner (week 7) while others may still be catching up on ROM. Adjust the program to the patient’s status – do not rush strengthening if ROM is still incomplete.  **Clinic visits:** If progress is good, therapy frequency might taper to 1x/week by week 8, focusing on checking form and progressing exercises, with patient largely independent in their workouts. |
| **Week 9–12** | **Pain:** Typically none or negligible. Patient may experience only occasional muscle soreness after strenuous activity.  **ROM:** Full ROM maintained (compare to opposite side; should be nearly symmetric in flexion/extension and forearm rotation).  **Strength:** Gradual return to baseline strength. By week 12, strength is often 90-100% of the uninvolved side for elbow motions (subjective and measured via manual testing or dynamometer).  **Function:** Patient should be functionally independent. Able to perform all usual ADLs and most work/sport tasks. Any remaining limitations are minor (perhaps slight stiffness after heavy use). | **Goal:** Achieve final strength gains and ensure a safe return to full function.  **Maximal strengthening:** In final weeks, push toward normal strength. Use higher resistances in exercises (as tolerated) aiming for last increments of strength. Include eccentric training for maximal strength (e.g. slow lowering of heavier weight than can lift up, under supervision).  **Plyometrics & power:** If appropriate to patient’s goals, include high-level plyometrics (clap push-ups, medicine ball rebounds) and power drills. These are typically for athletes or very active individuals and should be done only if elbow is pain-free and stable.  **Full functional retraining:** By now, patient transitions to activity-specific training. For athletes, begin controlled practice sessions of their sport with proper warm-up. For laborers, simulate full work demands in therapy (lifting heavy objects, using tools with force) to build confidence.  **Maintenance plan:** Develop a long-term exercise plan for the patient to continue post-discharge: a mix of stretching (to prevent recurrent tightness) and strengthening (to continue improving). Emphasize lifelong fitness of the arm to avoid future issues.  **Final assessment:** Perform outcome measures – e.g. goniometric ROM, strength testing, and functional scores (Mayo Elbow Performance Index, etc.) – to document improvements from baseline. Address any remaining deficits with targeted exercises in these last sessions. | **Independent gym program:** Patient should be transitioning to a self-managed routine. Encourage continuing strength workouts and stretching 2–3× weekly beyond therapy discharge to cement gains. Provide written instructions for any critical exercises.  **Stretching:** Advise the patient to integrate elbow stretches into their general fitness routine (especially if they notice morning stiffness or after heavy activity). E.g. a brief session of elbow ROM exercises daily or every other day.  **Home/work integration:** By 12 weeks, the patient is using the arm normally at home and work. They should continue to practice any challenging tasks at home to build more proficiency (for instance, practicing a musical instrument, gardening, or sport drills relevant to them).  **Self-monitoring:** Patient is taught to self-monitor for any signs of regression (increasing stiffness or pain) and to continue home exercises accordingly. | **Considerations:**  **Discharge planning:** Usually formal PT is concluded around 12 weeks post-op as patient meets goals​. Ensure patient has no pain with ADLs and full motion, and strength close to normal​.  **Follow-up:** Schedule a follow-up with the surgeon at 3 months to evaluate outcome; typically, by 3 months post-release, patients have achieved significant ROM gains (average ~30–40° arc improvement)​. If residual stiffness remains (e.g. >10° extension loss or functional limitation), the surgeon may consider interventions like a corticosteroid injection or manipulation under anesthesia​.  **Maintenance:** Instruct that improvements can continue up to 6–12 months post-op with persistent exercise​. The patient should maintain a home program to preserve gains. Even minor ROM improvements (a few degrees) beyond 3 months can enhance function and are worth pursuing​.  **Outcome:** By 12 weeks, most patients have markedly better elbow function and little to no pain​. They can resume full activities, though return to high-level sports or maximal strength might occur in subsequent months as they continue conditioning​ |

*Statistical analysis*

Patient data were securely coded and stored using the hospital’s electronic data capture system on local servers. The primary analysis was performed on the per-protocol population, with sensitivity analysis evaluated according to the intention-to-treat (ITT) principle. Missing data were handled using multiple imputation methods to ensure robust results. Prior to outcome analysis, baseline characteristics of the two groups were compared to ensure successful randomization; continuous variables were compared using Student’s t-tests (or Mann–Whitney U tests if assumptions of normality were violated) and categorical variables were compared using chi-square tests (or Fisher’s exact tests for expected cell counts <5). For key safety comparisons, Fisher’s exact test was specifically used to compare the proportion of patients experiencing at least one surgery-related adverse event in each group.

For the primary outcome of ROM, we used a linear mixed-effects model for repeated measures (MMRM) to assess differences in elbow ROM over time between the AR and OA groups. This model accounted for within-patient correlation across the three postoperative time points (6 weeks, 12 weeks, 1 year). The fixed effects included treatment group, time (as a categorical variable), and the interaction of group×time, and we adjusted for baseline ROM as well as the recorded patient treatment preference as covariates (including preference was exploratory, to account for any impact of patient expectation). From this model, we obtained the estimated between-group difference in ROM at 1 year (our primary endpoint of interest) with a 95% confidence interval and p-value. We similarly analyzed secondary continuous outcomes (ASES score, DASH score, strength measures) with mixed models, controlling for baseline values of those measures. For binary outcomes (e.g., whether a patient achieved a functional arc of motion ≥100°, or the occurrence of any adverse event), we used chi-square tests or Fisher’s exact test as appropriate. In particular, we used Fisher’s exact test to compare the proportion of patients with ≥1 surgery-related adverse event in each group, given some expected counts were low. The cost-effectiveness analysis involved calculating the total cost per patient (sum of all cost components) and then computing the incremental cost-effectiveness ratio (ICER) of AR vs OA in terms of cost per improvement in DASH score at 1 year [11]. Bias-corrected bootstrapping (5,000 resamples) was used to obtain confidence intervals for cost differences and ICER.

Dichotomous outcomes (such as achieving a functional motion arc by 1 year) were compared using the Cochran–Mantel–Haenszel chi-square test, controlling for baseline contracture severity category. A hierarchical testing strategy was prespecified for secondary outcomes to control multiple comparisons in a limited way. The secondary endpoints were ordered by clinical importance, and each was tested sequentially. If a given secondary outcome did not reach the significance threshold, subsequent secondary outcomes in the hierarchy were not considered statistically significant even if their p-values were below 0.05. In practice, secondary results are reported with point estimates and 95% confidence intervals unadjusted for multiple comparisons, and p-values are presented only up until the first non-significant result in the hierarchy. This approach preserves interpretability without formally inflating type I error across all secondary analyses.

One methodological consideration was the rounding of ROM measurements to the nearest 5°, which could introduce discrete increments in the data. To address any potential bias from this rounding, a correction to the significance level for ROM outcomes was applied as recommended by Zdravkovic and Jost [12]. Based on their method, the alpha level for hypothesis testing on the primary ROM outcomes was adjusted to 0.026 to account for the granularity of the data (details of this calculation are provided in the Supplemental Material). For all other outcomes, a two-tailed p < 0.05 was considered statistically significant. Statistical analyses were conducted using SPSS (v27, IBM) and R (v4.0).

**Results**

*Cost-effectiveness Analysis*

We evaluated costs from a combined health system and patient perspective over the 1-year postoperative period. This included direct hospital costs (surgery, implants, hospital stay), post-discharge medical costs (rehabilitation therapy, clinic visits in primary or secondary care), medication costs (including prophylactic and pain medications), and certain indirect patient costs (transportation for medical visits, etc.), as detailed in Table 5. The total average cost per patient did not significantly differ between the AR group (¥80,147.64 ± 18,494.78) and the OA group (¥78,416.03 ± 20,115.72; p=0.552). However, the cost composition varied notably between groups. The AR group had significantly lower physical therapy/rehabilitation costs on average (¥5,043 in AR vs ¥10,197 in OA, p<0.001), reflecting fewer outpatient PT sessions and shorter duration of therapy needed for AR patients. The AR group also had roughly half the primary care follow-up cost (¥901 vs ¥1,810, p<0.001), since fewer AR patients needed additional GP or local clinic visits for pain or wound checks (some OA patients saw their local doctor for wound care or stiffness). On the other hand, AR incurred higher intraoperative medication/supply costs (¥19,535 vs ¥10,978, p<0.001). This category included the arthroscopic disposable instruments, radiofrequency probes, and fluid management systems, which are costly, as well as slightly longer OR time cost. We also noted AR had a marginally higher postoperative medication cost (e.g., more patients took NSAIDs for HO prophylaxis); however, the biggest contributor was surgical equipment. Hospitalization costs (which include ward stay and OR time apart from instruments) were similar between groups (AR ¥5,805; OA ¥6,003; p=0.21) since length of stay (2 days) and surgical time were comparable. Other cost categories such as secondary specialist care (e.g., any surgeon follow-up beyond routine, which was rare) and patient non-medical costs (transportation, home care, etc.) did not differ significantly (Table 5). Combining all these, the slightly higher intra-op costs for AR were offset by significantly lower post-op rehab and follow-up costs, yielding nearly equal total costs.

From a value standpoint, we found that AR, with its better outcomes at roughly equal cost, was the more cost-effective strategy. The incremental cost-effectiveness ratio (ICER) for AR compared to OA was -¥1,527.5 per DASH point improved (95% CI: -¥7,332.5 to ¥4,107.1) at one year, meaning AR achieved greater clinical improvement at slightly lower or equal cost (a negative ICER indicates cost savings per unit improvement). We also computed the “net benefit” in terms of ROM gained per cost: AR had a slightly higher percentage of lost motion recovered per ¥10,000 spent, indicating a more efficient use of resources for ROM gain. These economic findings suggest that, in our setting, the arthroscopic approach provided better outcomes without adding financial burden, and in fact tended to reduce downstream costs (especially rehabilitation) enough to compensate for its higher operative expense. We have added clarifications in the text about the perspective (societal) and provided explanations for the differences in rehab and medication costs: specifically, the lower rehab cost in AR is attributed to quicker recovery and fewer therapy sessions required, while the higher “medication/implement” cost in AR stems from the specialized arthroscopic equipment and supplies.

*Adverse Events*

The incidence of adverse events was comparable between AR (31 of 96 patients, 32.3%) and OA (37 of 96 patients, 38.5%) groups (p=0.36) (Table 7). Most adverse events were minor and transient in nature, and importantly, all patients had resolution of these issues by final follow-up except those counted as serious (detailed below). The most common adverse events in both groups were temporary elbow pain or swelling exceeding the usual course, which occurred in about 15% of patients in each group. For instance, some patients experienced persistent swelling and stiffness in the first 4–6 weeks that required additional steroid injections (2 in AR vs 3 in OA) or extended therapy; these were logged as adverse events. Postoperative inflammation/pain requiring narcotics beyond 2 weeks was noted in 5 AR and 7 OA patients (managed with medication adjustments and extra PT, no long-term issues). Such events were evenly distributed (p=0.55).

No case of clinical heterotopic ossification requiring reoperation occurred in either group. There were no nerve injuries causing permanent motor deficits. However, we recorded a few transient neuropraxias: specifically, transient ulnar nerve numbness in 3 AR patients and 4 OA patients (in AR, likely from traction during arthroscopy; in OA, from surgical handling); all these cases resolved within 2–8 weeks with conservative management (observation and vitamin B supplementation). Two AR patients and one OA patient had transient median nerve neurapraxia (paresthesias in the thumb/index) which fully recovered by 3 months; these were possibly tourniquet or positioning related. Superficial wound complications were rare: only the OA group had 2 cases (2.1%) of superficial incision infection (one patient had mild erythema and drainage at the posterior incision, another had stitch abscess); both were successfully treated with oral antibiotics and local wound care, with no further issues. The arthroscopic portal sites in all AR patients healed uneventfully (0 superficial infections in AR).

Serious adverse events were infrequent. Deep infection requiring reoperation occurred in 2 patients (2.1%) in the OA group and in 0 patients in the AR group. The two OA deep infections were: one case of deep abscess and joint sepsis at 3 weeks post-op that necessitated surgical debridement and antibiotic beads, and another case of organ-space infection that required two washouts; both patients eventually recovered motion after aggressive therapy but were categorized as having experienced serious complications. There were no deep infections in AR, a notable difference (though with such low numbers, p=0.24 by Fisher’s test). Other SAEs included: hospitalization for manipulation under anesthesia (MUA) due to early re-stiffening – this occurred in 2 OA patients versus 1 AR patient. Those cases were considered “treatment failures” in some respect; after MUA at ~8 weeks post-op, they regained motion (and no further surgery was needed). There were no permanent nerve palsies; one AR patient developed complex regional pain syndrome (CRPS type I) characterized by pain and stiffness, which we treated with nerve blocks and intensive OT – this resolved by 1 year but was counted as serious due to prolonged rehabilitation and partial disability during that period. In total, 4 AR patients (4.2%) and 6 OA patients (6.3%) experienced a serious adverse event (p=0.75). All such patients were managed appropriately and, per protocol, were withdrawn from the formal therapy study (they continued standard care). There were no procedure-related systemic complications (no DVT, no anesthetic complications).

In summary, both techniques were reasonably safe, with a majority of complications being minor and transient. AR showed a trend towards a better safety profile (fewer infections and slightly fewer overall events), but statistically, the difference in overall complication rates was not significant in this sample. The notable qualitative difference was the absence of deep infections in the AR cohort, aligning with the inherently less invasive nature of arthroscopy (which avoids large incisions and exposure of bone, thereby reducing infection risk). All patients who experienced complications were successfully treated, and by the final follow-up, there was no significant difference in ultimate outcomes between those who had minor complications and those who did not (complication management largely prevented long-term detriment).

**References:**

1. Rai S, Zhang Q, Tamang N, Jin S, Wang H, Meng C. Arthroscopic arthrolysis of posttraumatic and nontraumatic elbow stiffness offers comparable clinical outcomes. BMC Musculoskelet Disord. 2019; 20:285.
2. Ball CM, Meunier M, Galatz LM, Calfee R, Yamaguchi K. Arthroscopic treatment of post-traumatic elbow contracture. J Shoulder Elbow Surg. 2002; 11:624-629.
3. Lapner PC, Leith JM, Regan WD. Arthroscopic debridement of the elbow for arthrofibrosis resulting from nondisplaced fracture of the radial head. Arthroscopy. 2005; 21:1492.
4. Blonna D, Wolf JM, Fitzsimmons JS, O’Driscoll SW. Prevention of nerve injury during arthroscopic capsulectomy of the elbow utilizing a safety-driven strategy. J Bone Joint Surg Am. 2013 Aug 7;95(15):1373-1381.
5. O’Driscoll SW, Blonna D. Osteocapsular Arthroplasty of the Elbow: Surgical Technique. JBJS Essent Surg Tech. 2013 Aug 14;3(3): e15.
6. Blonna D, O’Driscoll SW. Delayed-onset ulnar neuritis after release of elbow contracture: preventive strategies derived from a study of 563 cases. Arthroscopy. 2014;30(8):947-956.
7. Nobuta S, Sato K, Kasama F, Hatori M, Itoi E. Open elbow arthrolysis for post-traumatic elbow contracture. Ups J Med Sci. 2008;113(1):95-102.
8. King GJ, Richards RR, Zuckerman JD, Blasier R, Dillman C, Friedman RJ, et al. A standardized method for assessment of elbow function. Research Committee, American Shoulder and Elbow Surgeons. Journal of Shoulder & Elbow Surgery. 1999;8(4):351-4.
9. Jester A, Harth A, Wind G, Germann G, Sauerbier M. Disabilities of the arm, shoulder and hand (DASH) questionnaire: Determining functional activity profiles in patients with upper extremity disorders. J Hand Surg Br. 2005 Feb;30(1):23-8.
10. Morrey BF, Askew LJ, An KN, Chao EY. A biomechanical study of normal elbow motion. J Bone Joint Surg. 1981;63-A:872-827.
11. Circular of the Shanghai Municipal Bureau of Human Resources and Social Security on the Average Wages of Employed Persons in Full-Caliber Urban Units in the City in 2022. available: <https://rsj.sh.gov.cn/>.
12. Zdravkovic V, Jost B. Rounded data have a high potential for false comparative statistical results as shown with elbow arc of motion. J Shoulder Elbow Surg. 2018;27(2):276-81.
